# Efficacy and Safety of Ginkgo Biloba Pills for Coronary Heart Disease with Impaired Glucose Regulation: Study Protocol for a Series of* N*-of-1 Randomized, Double-Blind, Placebo-Controlled Trials

**DOI:** 10.1155/2018/7571629

**Published:** 2018-10-14

**Authors:** Mingyue Sun, Lulu Chai, Fang Lu, Yang Zhao, Qingna Li, Boya Cui, Rui Gao, Yue Liu

**Affiliations:** ^1^Institute of Clinical Pharmacology of Xiyuan Hospital, China Academy of Chinese Medical Sciences, Beijing 100091, China; ^2^Cardiovascular Diseases Center, Xiyuan Hospital of China Academy of Chinese Medical Sciences, Beijing 100091, China

## Abstract

**Background:**

Coronary heart disease has become a serious challenge to China with its high prevalence and mortality. The impaired glucose regulation is prevalent in patients with cardiovascular disease. However, there are few drugs that interfere early with impaired glucose regulation. Ginkgo biloba extract not only is a commonly used drug for cardiovascular diseases, but also has a significant effect in reducing blood sugar. Therefore, this study used a single-case randomized controlled trial to explore the efficacy of Ginkgo biloba pills in the treatment of coronary heart disease patients with impaired glucose regulation.

**Methods/Design:**

This is a randomized, double-blind, placebo-controlled, three-period crossover trial for a single subject. A total of 12 subjects will be recruited in this trial. The trial is divided into three cycles, and one cycle has two treatment periods. Ginkgo biloba pills and placebo will be randomized during the treatment period. The test period will last for 58 weeks and subjects will take 48 weeks. Subjects will be selected by the researcher strictly in accordance with the inclusion and exclusion criteria.

**Discussion:**

Ginkgo biloba preparations are widely used in cardiovascular diseases both at home and abroad due to their definite curative effect, few side effects, various dosage forms, and convenient and safe use. Diabetes mellitus is a high-risk factor for the occurrence of cardiovascular disease. Therefore, it is of great significance to control the impaired glucose regulation and slow down the development of diabetes and reduce the incidence and mortality of cardiovascular diseases. This trial is registered with ClinicalTrials.gov (ID: NCT03483779).

## 1. Background

With the development of economy and the improvement of living standards, the prevalence and mortality of coronary atherosclerotic heart disease (CHD) have been significantly higher than before and have been more in younger age in China. The global disease burden report [1], which was published in the Lancet in 2015, showed CHD ranked second in the cause of death in the Chinese population in 2013. Therefore, cardiovascular disease is a serious challenge for China's public health and health care.

As we all know, diabetes is one of the important independent dangerous factors for CHD, and almost all patients with type 2 diabetes experience a prediabetic stage, which is usually characterized by impaired fasting glucose, impaired glucose tolerance, etc. This stage is medically known as impaired glucose regulation (IGR). The crowd at this stage is characterized by blood glucose levels that are already above the normal range, but have not yet met the criteria for diabetes. At the European Society of Cardiology Annual Meeting 2004, the European Heart Survey released the first results of a survey on the state of glucose metabolism in patients with CHD. Studies have shown that about two-thirds of patients with CHD have diabetes or IGR [2]. The findings further demonstrate the cross-coexistence relationship between diabetes and cardiovascular disease and also indicate that IGR is prevalent in patients with cardiovascular disease. The U.S. Bureau of Health Care Research and Quality conducted a systematic review of 156 prediabetes studies [3], suggesting that prediabetes is associated with a higher risk of diabetes and cardiovascular disease, and early identification and treatment of prediabetic patients may reduce or delay the progression of diabetes and its associated cardiovascular complications and microvascular disease. Therefore, we should take effective measures to intervene in prediabetes in a timely manner.

At present, early treatment of IGR is mainly based on lifestyle interventions, including health education, diet therapy, exercise therapy, weight control, and smoking cessation and abstinence. However, lifestyle interventions in the actual living environment are effective, but poor patient compliance and clinically continuous and strict follow-up often fail to achieve the desired effect [4]. Some clinical trials have also confirmed the effectiveness of drug interventions such as acarbose, metformin, and other drugs [5]. The drugs selected for IGR should meet the requirements of long-term use, no serious adverse reactions, no hypoglycemia, and other conditions, but, at present, there are few related drug researches, and the choice of drugs is relatively limited in clinical applications.

## 2. Ginkgo Biloba

Ginkgo biloba is a leaf of Ginkgo biloba L., which has attracted much attention due to its high medicinal value. In traditional Chinese medicine, Ginkgo biloba is considered to be flat, sweet, bitter, and astringent, and it has the function of promoting blood circulation and removing blood stasis, removing obstruction in the channels to stop pain, astringing Lung-Qi and relieving cough and melting turbidity and reducing lipid decoction. It is commonly used in the treatment of obstruction of collaterals by blood stasis, chest stuffiness and pains, apoplectic hemiplegia, etc. [6]. Since the 1960s, with the in-depth study of the chemical constituents of Ginkgo biloba by domestic and foreign scholars, their pharmacological effects and clinical applications have been under further understanding.

Related studies have shown that the effective medicinal components extracted from Ginkgo biloba are mainly flavonoids, terpene lactones, and organic acids, which increase blood flow, prevent blood clots, reduce blood lipids, regulate the central nervous system, improve memory function, and improve cognitive function. The known pharmacological effects are mainly related to the mechanisms of antagonizing platelet activating factor (PAF), antioxidation, scavenging free radicals, and regulating the release of excitatory amino acids [7]. Current research shows that insulin resistance and insulin secretion deficiency are the main pathogeneses of diabetes, and Ginkgo biloba extract (EGb) has a bidirectional role in regulating insulin secretion. On the one hand, it can improve hyperinsulinemia in patients with insulin resistance. On the other hand, it can increase insulin secretion in patients with impaired islet function, and its mechanism of action is mainly to protect *β*-cells, promote insulin secretion, reduce free fatty acid (FFA) levels, inhibit activation of inflammatory factors, increase adiponectin, decrease leptin levels, and activate glucose transporter 4 to improve insulin resistance [8].

More studies [9] confirmed the hypoglycemic effect of EGb. Daye Cheng et al. [10] administered streptozotocin-induced diabetic rats with high, medium, and low doses of EGb. After 30 days of treatment, the blood glucose level in the different doses of EGb treatment group was significantly lower than that in the control group. The blood glucose level in the high-dose EGb group decreased to the normal range, and its hypoglycemic effect was significantly dose and time dependent. Clinical studies have found that EGb can improve insulin resistance in patients with CHD and have the effect of regulating blood glucose and lipid metabolism [11]. Studies of EGb intervening in db/db diabetic mice have shown that EGb can significantly reduce fasting blood glucose in db/db diabetic mice by increasing the islet mass, reducing the level of oxidative stress in the pancreatic islets, significantly reducing fasting blood glucose in db/db diabetic mice, improving insulin sensitivity, and improving glucose tolerance and beta-cell secretion, especially early phase insulin secretion [12]. Our previous study also found that EGb pretreatment can reduce myocardial injury induced by acute myocardial ischemia in type 2 diabetic rats, and its cardiovascular protection may be closely related to the activation of actin clearance system [13].

## 3. Why N-of-1 Trials

The single-case randomized controlled (N-of-1) trials were designed for a single patient or a series of individual patients, with multiple prospective clinical randomized crossover trials alternating between multiple trials and controls [14]. Professor Gordon of McMaster University in Canada pointed out that in the latest evidence pyramid, the single-case randomized controlled trial is at the highest level of clinical research evidence, and the systematic review of randomized controlled trials is in second place [15]. N-of-1 trial is a clinical efficacy evaluation method that meets the self-regulation and characteristics of the individual medical treatment in traditional Chinese medicine (TCM). The study of N-of-1 for TCM can highlight individualized diagnosis and treatment and maintain TCM characteristics. It can also make the clinical research of TCM more scientific and can avoid the influence of subjective selection, implementation and measurement bias, and individual differences, especially for chronic diseases requiring long-term treatment. Therefore, in this study, patients with CHD and IGR will be selected as the study subjects. N-of-1 trials will be used to evaluate the clinical efficacy of Ginkgo biloba pills and to explore the individualized therapeutic evaluation methods applicable to TCM research.

## 4. Methods

### 4.1. Trial Design

This is a randomized, double-blind, placebo-controlled, single-case clinical trial in which each subject can be considered as having its own RCT. Twelve subjects with CHD associated with IGR will be screened. Each N-of-1 trial will consist of 3 cycles, including 6 treatment periods, 2 of which are in one group, including 8 weeks of placebo treatment and 8 weeks of Ginkgo biloba. Each subject took Ginkgo biloba drop pills or Ginkgo biloba drop pills simulant at random. There was no washout period at the beginning of the study and there was a 2-week washout period before each treatment period. The entire experimental observation time window is 58 weeks ± 5 days.  Table 1 shows the study design of the subject's medication process. The protocol conforms to the Standard Protocol Items: CONSORT extension for reporting N-of-1 trials (CENT) 2015 [16]. The CENT 2015 checklist can be found in Additional file 2.

### 4.2. Study Participants

All patients participating in the experiment should be screened strictly in accordance with the following criteria.

#### 4.2.1. Diagnostic Criteria

*(1) Diagnostic Criteria for Coronary Heart Disease*. According to the 2010 Guidelines for the Diagnosis and Treatment of Clinical Coronary Heart Diseases.

(a) Clear history of old myocardial infarction, or a history of PCI, or a history of CABG (at least 3 months).

(b) Coronary angiography or coronary CTA results suggesting that at least one coronary branch is stenotic and the degree of stenosis is ≥50%.

*(2) Diagnostic Criteria for Stable Angina*. According to the Chinese Medical Association, Cardiovascular Branch, the Chinese Journal of Cardiovascular Diseases Editors Committee 2007 Guidelines for the Diagnosis and Treatment of Chronic Stable Angina.

The main content is that when there is physical activity or mental stress, coronary blood flow cannot meet the needs of myocardial metabolism, leading to myocardial ischemia-induced angina pectoris, and rest or nitroglycerin can be relieved. Chronic stable angina patient is a patient whose degree, frequency, nature, and predisposing factors of angina pectoris have not changed significantly within 1 to 3 months.

*(3) Impaired Glucose Regulation (Prediabetes) Diagnostic Criteria*. According to the 2016 American Diabetes Association (ADA) diagnostic criteria: impaired fasting glucose regulation (IFG); fasting blood glucose (FPG) 5.6 ~ 6.9mmol/L, or glucose tolerance decrease (IGT); 2 h blood glucose (2hPG) 7.8 to 11.0mmol/L after oral administration of 75g glucose in OGTT, or glycosylated hemoglobin (HbA1c) in 5.7% to 6.4 %.

*(4) Diagnostic Criteria for Blood Stasis Syndrome*. According to the “Diagnostic Criteria for Coronary Heart Disease and Blood Stasis Syndrome” promulgated by the Professional Committee for the Promotion of Blood and Blood Stasis of the Chinese Association of Integrative Medicine in China in 2016.

#### 4.2.2. Inclusion Criteria

Participants fulfill the following inclusion criteria: (1) male and female patients with clear history of previous myocardial infarction or history of percutaneous coronary intervention (PCI) or history of coronary artery bypass grafting (CABG) (at least 3 months or more), or who have coronary angiography or coronary CT angiography (CTA) results suggesting at least one coronary artery stenosis and lumen stenosis ≥ 50%, (2) in line with the criteria for stable angina, and the number of episodes of angina pectoris ≥ 2 times per week, (3) complying with the diagnostic criteria of blood stasis syndrome of coronary heart disease (CHD), (4) complying with the 2016 Diabetes Association (ADA) published criteria for impaired diagnosis of glucose regulation, and (5) aging between 18 and 75 years. Participants voluntarily participated in this study, signed informed consent, and had good compliance.

#### 4.2.3. Exclusion Criteria

Patients who meet any of the following will be excluded: (1) with congenital or rheumatic heart disease or severe cardiopulmonary insufficiency (grades 3 and 4 of cardiac function), or uncontrolled severe arrhythmias (including ventricular tachycardia, supraventricular tachycardia), or not controlled hypertension (systolic blood pressure ≥ 160 mmHg or diastolic blood pressure ≥ 100 mmHg), (2) with cerebrovascular disease, or with severe liver and kidney dysfunction, or with endocrine, urinary, blood system, and other serious primary diseases, (3) within 4 weeks, having a history of major organ surgery such as head, chest, or abdomen or bleeding tendency, (4) those who have taken hypoglycaemic agents or glucocorticoids, thiazide diuretics, and other drugs that affect blood sugar levels within 3 months, (5) people with diseases affecting blood glucose metabolism, such as thyroid glands and adrenal diseases, or those with previous history of the aforementioned diseases, (6) allergies or persons allergic to known ingredients of the studied drug, (7) pregnant and lactating women or those with a pregnancy plan, and (8) subjects who participated in other clinical trials in the last 3 months. Researchers consider that subjects should not participate in clinical trials.

### 4.3. Interventions

All subjects participating in the trial will continue to receive basic medicine. Subjects will take random oral Ginkgo biloba pills or Ginkgo biloba drop pills simulant at each period, three times a day, five at a time.

### 4.4. Drug Inventory

At each follow-up visit, the drug administrator will record the amount of the drug accepted, taken, and returned by the subject as a matter of fact to determine the adherence of the subject and record it in a timely manner. Subject medication compliance = (dosage amount - not used)/dose should be×100%.

### 4.5. Randomization

**Figure 1:**
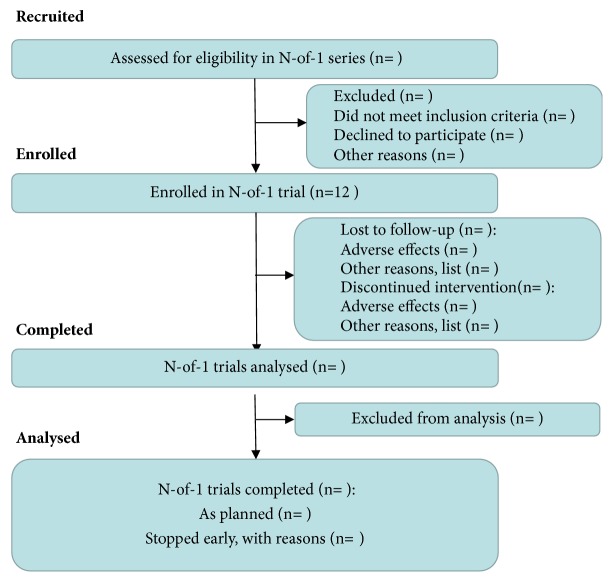
Flow of participants through the study.

With the help of statisticians at Xiyuan Hospital of China Academy of Chinese Medical Sciences, this test will use block randomization, and with a block size of 2. The sequence of test drugs is applied by the SAS random program to generate a “test drug random coding table.” The test drugs were randomly assigned by the database administrator according to the coding table. The prepared research drugs will be delivered, stored, and distributed in the pharmacy of Xiyuan Hospital Clinical Pharmacology Institute. Patients and investigators were blinded to all randomization and packaging procedures until completion of trial. And each subject was randomly assigned to three matched treatment groups, such as BA-AB-BA or AB-BA-BA, in the order in which they were treated.  Figure 1 shows the flow of participants through the trial.

### 4.6. Blinding

During the trial, the blinding method will be strictly applied to the investigator and the subject. The personnel who did not directly participate in the trial will be randomly assigned to the subject to give the drug. Placebo was used to screen placebos that most closely resembled the appearance, odor, and taste of Ginkgo biloba pills, and placebos were packaged in the same way as Ginkgo biloba pills to minimize the risk of blindness. The experiment was designed with a two-level blind method and blinded concealment was performed at the same time. The first-level blind-end blindness was achieved after researchers completed clinical trials and statistical analysts completed data management. After completing the statistical analysis and obtaining the corresponding conclusions, the secondary blind will be uncovered and eventually form a statistical analysis report.

### 4.7. Combination Therapy

No other blood circulation and blood stasis herbs and hypoglycemic drugs can be taken at the same time during the test. If the patient has existing underlying diseases, such as hypertension and hyperlipidemia, they can be combined according to the relevant guidelines. If angina occurs during the trial, nitroglycerin can be given and the dose, number of uses, and time can be recorded in the case report form.

The medicines that must be taken in conjunction with the disease can remain unchanged. If there are other treatments, the medicine name or other treatment name, dosage, number of uses, and time must be recorded in the case report form.

### 4.8. Efficacy Index

Subjects need to be at baseline, treatment week 8, week 10, week 18, week 20, week 28, week 30, week 38, week 40, week 48, week 50, and week 58 visits. Each visit requires observation and recording of efficacy indicators.

The main evaluation indicators for this trial are the changes in blood glucose and the Seattle Angina Questionnaire (SAQ). The measurement of blood glucose mainly observed the changes of fasting plasma glucose (FPG) and 2 h postprandial blood glucose (2hPG).

The secondary evaluation criteria will be mainly observing the following changes: the incidence of angina symptom scores, C-reactive protein, glycosylated hemoglobin (HbA1c), fasting insulin, blood lipids, metabolomics analysis, and major adverse cardiac and cerebral vascular events (MACCE).

### 4.9. Outcomes

*(1) Primary Outcomes*

*(a) The Changes in Blood Glucose*. The value of fasting blood glucose (FPG) and the value of 2 h postprandial blood glucose (2hPG).

*(b) The Seattle Angina Questionnaire (SAQ)*. The SAQ dose judgment criteria are as follows. The table is divided into 5 items and 19 items: physical activity limitation (PL, question l), angina stable state (AS, question 2), angina pectoris (AF, questions 3-4), treatment satisfaction (TS questions 5-8), and disease cognition (DP, questions 9-11). After scoring the 19 items of the 5 major items one by one, each score is forwarded.

The standard score = (actual score - the lowest score in this aspect)/(the highest score in this aspect - the lowest score in this aspect)×100; the higher the score, the better the quality of life and the functional state of the patient.

*(2) Secondary Outcomes*. They are angina symptom score, C-reactive protein, glycosylated hemoglobin (HbA1c), fasting insulin, blood lipids, metabolomics analysis, and the incidence of composite endpoints of major adverse cardiac and cerebral vascular events (MACCE).

Blood lipids are as follows: cholesterol, triglycerides, high-density lipoprotein, and low-density lipoprotein.

Metabolomics test and analysis: 1 ml of plasma, 2 ml of serum, 5 ml of urine, and 10 g of feces were collected each time. Metabolomic analysis of blood samples, feces, and urine samples was performed to analyze the differences in metabolites between patients taking Ginkgo biloba drop pills and placebo before and after treatment.

Major adverse cardiac and cerebral vascular events (MACCE) include cardiovascular death, nonfatal myocardial infarction, stent thrombosis, revascularization, ischemic stroke, and hospitalization for unstable angina.

### 4.10. Safety Indicators

The safety indicators will include the occurrence of adverse events and serious adverse events, vital signs, and laboratory tests. The subjects were required to have their examination records at baseline, 8 weeks, and 58 weeks of treatment, except for the baseline test during the pregnancy test.

(1) Adverse events and serious adverse events: statistical analysis of the occurrence of adverse events and serious adverse events.

(2) Vital Signs: analysis of blood pressure, respiration, heart rate, and body temperature before and after treatment.

(3) Laboratory tests: including blood routine, urine routine, liver function, renal function, blood lipids, 12-lead ECG, and urine pregnancy test for women of childbearing age; analyzing laboratory tests before and after the test for abnormalities, clinical significance, and test drug relevance.

### 4.11. Adverse Events

*(1) Adverse Events (AE)*. Definition: A patient or clinical trial recipient receives a drug after an adverse medical event but does not necessarily have a causal relationship with the treatment. Thus, an adverse event refers to any unanticipated signs associated with the use of a medical (study) product, including laboratory abnormalities, symptoms, or conditions, whether or not medical (study) products are considered. Thus, an adverse event refers to any unanticipated signs associated with the use of a medical (study) product, including laboratory abnormalities, symptoms, or conditions, whether or not medical (study) products are considered.

*(2) Serious Adverse Events (SAE)*. Definition: Severe adverse events refer to the following adverse events at any dose of the test drug or at any time during the observation period, including hospitalization, extended hospital stay, disability, work ability, life-threats or death, and congenital malformations.

*(3) Judgment of Causal Relationship between Adverse Events and Test Drugs*. Adverse reaction evaluation criteria (including symptoms, signs, and laboratory indicators) are as follows.

(1) The time of adverse reactions coincides with the time of administration.

(2) Adverse reactions are associated with known adverse effects of the drug.

(3) Adverse reactions cannot be explained by other reasons.

(4) The adverse reactions are alleviated or disappeared after stopping the drug.

(5) Adverse reactions are reproduced after administration.

*(4) Judgment Criteria for Adverse Events and Drug Relationships*. (1) It is definitely related to: the reaction appears in a reasonable time sequence according to the medication, and it is consistent with the type of reaction known to the suspected drug. The reaction disappears after the suspected drug is stopped, and the patient's clinical state or other reasons are unlikely to produce the reaction.

(2) It may be related to: the reaction appears in a reasonable time sequence according to the medication, and it is consistent with the type of reaction known to the suspected drug. The reaction can be alleviated after the suspected drug is stopped, but the patient's clinical state or other reasons may also produce the reaction.

(3) It may be irrelevant: the reaction appears to be inconsistent with the reasonable time sequence after administration and does not meet the type of response known to the suspected drug. The response is not alleviated after the suspected drug is discontinued, and the patient's clinical state or other causes may cause the reaction. The response is reduced after the state is improved or after other reasons are eliminated.

(4) It must be irrelevant: the reaction does not meet the reasonable time sequence after administration and does not meet the type of reaction known to the suspected drug. The clinical state of the patient or other reasons can produce the reaction, and the response is relieved after the disease state is improved or other causes are eliminated.

(5) It cannot be judged that there is no clear relationship between the occurrence of the reaction and the time sequence after administration, and the type of reaction known to the drug is similar, and other drugs used at the same time may cause the same reaction.

It is certain that it may be relevant, may be related, and cannot determine three adverse reactions that can be considered as drugs.

*(5) Observation and Recording of Adverse Events*. Subjects with AE should be followed up until the AE recovers (returns to normal or to baseline), or until the condition is stable, or until there is a reasonable explanation.

For AEs that have not recovered at the end of the observation period or AEs that were known at the last visit during the observation period, the investigator should follow up after the visit to return to the above satisfactory results. The necessary treatment, reports, and records should be given as required. For any exceptions, you must go through the consent of the sponsor's security management contact.

*(6) Records and Reports of Serious Adverse Events*. In case of serious adverse events during the trial, the unit undertaking the clinical trial shall immediately take the necessary measures to protect the safety of the subject and report to the local provincial drug regulatory authority and the State Food and Drug Administration, the sponsor within 24 hours. The clinical trial is responsible for the unit ethics committee and informs the participating units. The sponsor will ensure that the reporting procedures for all legal and regulatory requirements are met.

The SAE collection time for this study ranged from 30 days after the signed informed consent to the last use of the study drug.

### 4.12. Baseline

The baseline data will include demographic data: age, gender, height, weight, ethnicity, and occupation and general clinical data: history, duration, physical examination, comorbidity and medication, and start of trial date.

### 4.13. Statistical Methods

Statistical analysis software will use SAS statistical analysis software version 9.4.

The results were analyzed at the end of the test. The description of the quantitative indicators will calculate the mean, standard deviation, median, minimum, maximum, and interquartile range. The description of qualitative indicators will calculate the number of cases and percentage of each category. All statistical tests use two-sided tests. When the* P* value is less than or equal to 0.05, the difference is considered statistically significant (except where specifically stated). Descriptive statistics will be performed on the completion of the study. Demographics and baseline characteristics will be compared between groups to analyze the balance between the groups.

The analysis of efficacy indicators will use Revman5.0 software for meta-analysis.

### 4.14. Data Management

All the data comes from the research medical record, which is filled in by the researcher. The data was collected through the electronic case report form (e-CRF). Each selected case must complete the case report form. The data manager is responsible for the data management and establishes a dedicated database for data entry and management. Data entry is done by trained personnel and data quality and accuracy checks are performed. To ensure the accuracy of the data, two data administrators should independently perform double entry and proofreading.

## 5. Discussion

A single-case randomized controlled trial used a single subject as the study subject. Under the same specified conditions, the differences between different individuals were observed. This was in line with the differences between Chinese medicine and syndrome differentiation. Impaired sugar regulation is generally rarely treated in clinical practice. Doctors generally recommend that patients actively engage in various lifestyle interventions in order to prevent the rise of blood glucose. If the patient does not intervene or the intervention fails, which leads to the rise of blood glucose to diabetes, it worsens the patient's pain and condition. This study selected patients with coronary heart disease and IGR as research subjects to evaluate the efficacy of Ginkgo biloba pills in the treatment of coronary heart disease with glucose regulation. Patients with this disease can be provided with a new treatment idea. This study can be viewed as an exploratory study, using small samples to conduct experiments, thus saving manpower, material resources, and financial resources.

## Figures and Tables

**Table 1:** N-of-1 test for the treatment of coronary heart disease with impaired glucose regulation using Ginkgo biloba drop pills and simulant.

**Study Period**										

		**Enrolment**	**Allocation**	**Post allocation**						**Close-out**

				**Cycle 1**		**Cycle 2**		**Cycle 3**		

	**Time Point**	**D-7 to D-0**	**0**	**Weeks 1-10**	**Weeks 11-20**	**Weeks 21-30**	**Weeks 31-40**	**Weeks 41-50**	**Weeks 51-58**	**Week 58**

**Enrollment**	**Eligibility** **Screen**	×								
	**Informed consent**	×								
	**Allocation**		×							

**Intervention**	**Intervention Ginkgo biloba drop pills or Ginkgo biloba drop pills simulant**			×	×	×	×	×	×	×

**Assessment**	**Baseline variables**	×								
	**Urine pregnancy test for women of childbearing age**	×								
	**Physical examination**	×								×
	**Blood routine, urine routine, liver function, renal function, vital signs, ECG**	×		×						×
	**Blood glucose, blood lipids, SAQ, the angina symptom score, C-reactive protein**	×		×	×	×	×	×	×	×

*Note*. The trial consisted of three cycles and was divided into six treatment periods. Patients receiving Ginkgo biloba drop pills and placebo treatment were randomized. There are 8 weeks for each treatment period and a 2-week washout period at the end of treatment.

## Data Availability

The data used to support the findings of this study are included within the article and the protocol has been registered in ClinicalTrials.gov (No. NCT03483779).

## Supplementary Materials

Supplementary 1 Additional File 1: Diagnostic criterias and score scale.

Supplementary 2 Additional File 2: CENT 2015 checklist. The CENT 2015 checklist is an extension of 14 items of the 25 CONSORT 2010 items, providing detailed descriptions for some trials.

Supplementary 3 Additional file 3: Case report form.

Supplementary 4 Additional file 4: Informed consent form.
