# Supplementary material for: Efficacy and Safety of Ginkgo Biloba Pills for Coronary Heart Disease with Impaired Glucose Regulation: Study Protocol for a Series of N-of-1 Randomized, Double-Blind, Placebo-Controlled Trials
Source: Evid Based Complement Alternat Med. 2018 Oct 14;2018:7571629. doi: 10.1155/2018/7571629 (PMC6204161; doi:10.1155/2018/7571629)
Supplement: Supplementary 1 — Additional File 1: Diagnostic criterias and score scale. [file 7571629.f1.pdf]

Diagnostic criterias for the diagnostic criteria of coronary heart disease and blood stasis syndrome (BBS)

| Indicator type | Indicator content | Indicator assignment |
|---|---|---|
| Main indicator | 1．Chest pain fixed position | 10 |
| | 2．Tongue color purple or dark | 10 |
| | 3．The tongue has ecchymosis | 10 |
| | 4．Coronary angiography shows at least one coronary artery stenosis ≥ 75% | 9 |
| | 5．Ultrasound or angiography shows coronary thrombosis or intracardiac wall thrombosis | 8 |
| Secondary indicator | 1. Chest pain aggravated at night | 6 |
| | 2. The lips or gums are in a dark state | 7 |
| | 3. Sublingual varicose veins or dark purple | 7 |
| | 4. Coronary angiography shows at least one coronary artery stenosis ≥50%, but <75% | 6 |
| | 5. Partial thromboplastin time (APTT) or prothrombin time (PT) shortened | 5 |
| Auxiliary indicator | 1．Dark complexion | 2 |
| | 2．unsmooth pulse | 4 |
| | 3．Coronary CTA or coronary angiography shows significant calcification or diffuse lesions | 3 |
| | 4．Fibrinogen elevation | 3 |

Note:(1)meet the diagnostic criteria for coronary heart disease, need to meet: coronary angiography shows at least one coronary artery stenosis ≥ 50%;(2) coronary heart disease blood stasis syndrome score ≥ 19 points can be diagnos

ed as blood stasis syndrome, score the level of blood stasis can be used to evaluate the degree of blood stasis syndrome in coronary heart disease; (3)the diagnosis of coronary heart disease with blood stasis syndrome must include at least one macro indicator of the main index and the secondary index, and the simple physical and chemical indicators cannot be diagnosed.

Angina symptom score scale:

| Symptom | Score | | | |
|---|---|---|---|---|
| | 0 | 2 | 4 | 6 |
| Number of angina attacks | No | 2-6 episodes per week | 1-3 times a day | more than 4 episodes per day |
| Angina duration | No | Each time the pain lasts less than or equal to 5 minutes | Each time the pain lasts longer than 5 minutes and less than 10 minutes | Each time the pain lasts for 10 minutes or more |
| Angina pain level | No | Physical activity that is heavier than daily activities causes angina and daily activities are asymptomatic. Activities that can cause angina, such as trotting on the ground, fast or holding heavy objects on the third floor, up steep slopes, etc. | Daily physical activity causes angina and daily activities are slightly limited. Activities that can cause angina, such as walking 3-4 stations (3-4 miles) at normal speed under normal conditions, on the third floor, uphill, etc. | Lighter physical activity than daily activities causes angina and daily activities are significantly limited. Activities that can cause angina, such as walking 1-2 stations (1-2 miles) at normal speed under normal conditions, on the second floor, gentle slope, etc. |
| Nitroglycerin dosag | No | 1-4 tablets per | 5-9tablets per week | Take more than 10 tablets |

| | | | | |
|---|---|---|---|---|
| e | | week | | a  week |
