# Supplementary material for: Efficacy and Safety of Ginkgo Biloba Pills for Coronary Heart Disease with Impaired Glucose Regulation: Study Protocol for a Series of N-of-1 Randomized, Double-Blind, Placebo-Controlled Trials
Source: Evid Based Complement Alternat Med. 2018 Oct 14;2018:7571629. doi: 10.1155/2018/7571629 (PMC6204161; doi:10.1155/2018/7571629)
Supplement: Supplementary 2 — Additional File 2: CENT 2015 checklist. The CENT 2015 checklist is an extension of 14 items of the 25 CONSORT 2010 items, providing detailed descriptions for some trials. [file 7571629.f2.pdf]

CENT 2015 checklist

Section/Topic	No	Item	Page in article
title and abstract	1a	Identify as an ‘‘N-of-1 trial’’ in the title<br>For series; Identify as ‘‘a series of N-of-1 trials’ ’in the title	1
	1b	For specific guidance, see CENT guidance for abstracts	1-2
Introduction	2a.1	Scientific background and explanation of rationale	2-4
Background and	2a.2	Rationale for using N-of-1 approach	4
Objectives	2b	Specific objectives or hypotheses	4
Methods<br>Trial design	3a	Describe trial design, planned number of periods, and duration of each period(including run-in and wash out, if applicable)In addition for series; Whether and how the design was individualized to each participant, and explain the series design	4
	3b	Important changes to methods after trial start(such as eligibility criteria),with reasons	/
Participant(s)	4a	Diagnosis or disorder, diagnostic criteria comorbid conditions, and concurrent therapies	5-7
	4b	Settings and locations where the data were collected.	/
	4c	Whether the trial(s) represents a research study and if so, whether institutional ethics approval was obtained.	13
Interventions	5	The interventions for each period with sufficient details to allow replication, including how and when they were actually administered	7
Outcomes	6a.1	Completely defined pre-specified primary and secondary outcome measures, including how and when they were assessed	9-10
	6a.2	Description and measurement properties (validity and reliability) of outcome assessment tools	9-10
	6b	Any changes to trial outcomes after the trial commenced, with reasons	/
Sample size	7a	How sample size was determined	/
	7b	When applicable, explanation of any interim analyses and stopping guidelines	/
Randomization<br>Sequence generation	8a	Whether the order of treatment periods was randomized, with rationale, and method used to generate allocation sequence	7
	8b	When applicable, type of randomization; details of any restrictions (such as pairs, blocking)	7
	8c	Full, intended sequence of periods.	7

Allocation concealment mechanism	9	Mechanism used to implement the random allocation sequence (such as sequentially numbered containers), describing any steps taken to conceal the sequence until interventions were assigned	7
Implementation	10	Who generated the random allocation sequence, who enrolled participants, and who assigned participants to interventions	/
Blinding	11a	If done, who was blinded after assignment to interventions (for example, participants, care providers, those assessing outcomes) and how	8
	11b	If relevant, description of the similarity of interventions	8
Statistical methods	12a	Methods used to summarize data and compare interventions for primary and secondary outcomes	14
	12b	For series; If done, methods of quantitative synthesis of individual trial data, including subgroup analyses, adjusted analyses, and how heterogeneity between participants was assessed (for specific guidance on reporting syntheses of multiple trials, please consult the PRISMA Statement)	/
	12c	Statistical methods used to account for carry over effect, period effects, and intra-subject correlation.	/
Results<br>Participant flow (a diagram is strongly recommended)	13a.1	Number and sequence of periods completed, and any changes from original plan with reasons	/
	13a.2	For series; The number of participants who were enrolled, assigned to interventions, and analysed for the primary outcome	/
	13b	For each group, losses and exclusions after randomization, together with reasons	/
Recruitment	14a	Dates defining the periods of recruitment and follow-up	/
	14b	Whether any periods were stopped early and/or whether trial was stopped early, with reason(s)	/
Baseline data	15	A table showing baseline demographic and clinical characteristics for each group	/
Numbers analysed	16	For each intervention, number of periods analysed.<br>In addition for series; If quantitative synthesis was performed, number of trials for which data was synthesized	/
Outcomes and estimation	17a.1	For each primary and secondary outcome, results for each period; an accompanying figure displaying the trial data is recommended	/
	17a.2	For each primary and secondary outcome, the estimated effect size and its precision (such as95% confidence interval).In addition for series; If quantitative synthesis was performed, group estimates	/

		of effect and precision for each primary and secondary outcome	
	17b	For binary outcomes, presentation of both absolute and relative effect sizes is recommended	/
Ancillary analyses	18	Results of any other analyses performed,, including assessment of carryover effects ,period effects, intra-subject correlation	/
		In addition for series; If done, results of subgroup or sensitivity analyses	
Harms	19	All harms or unintended effects for each intervention. (for specific guidance see CONSORT for harms)	/
Discussion Limitations	20	Trial limitations, addressing sources of potential bias, imprecision, and, if relevant, multiplicity of analyses	/
Generalisability	21	Generalizability (external validity, applicability)of the trial findings	/
Interpretation	22	Interpretation consistent with results, balancing benefits and harms, and considering other relevant evidence	/
Other information Registration	23	Registration number and name of trial registry	/
Protocol	24	Where the full trial protocol can be accessed, if available	1
Funding	25	Sources of funding and other support (such as supply of drugs), role of funders	/

CENT abstract considerations

Item	Extension for N-of-1 designs	Page in article
Title	Identification of the study as an N-of-1 trial or series ofN-of-1 trials in the title	1
Authors	Contact details for the corresponding author	1
Trial design	Description of trial design, number of periods, and period duration	4
Methods		
Participant(s)	For individual trial, clinical condition under study<br>For series, eligibility criteria for participants	5-7
Interventions	Interventions intended for each period	7
Objective	Specific objective or hypothesis	4
Outcome	Clearly defined primary outcome for this report	9-10
Randomization	How participants were allocated to interventions	7
Blinding (masking)	Whether participant(s), care givers, and those assessing the outcomes were blinded to group assignment	8
Results;		
Numbers randomized	For individual N-of-1 trial, the number and sequence of periods completed<br>For series, number of individual trials carried out	4
Recruitment	Not applicable	
Numbers Analysed	For individual N-of-1 report, number of periods analysed for each intervention<br>For series, the number of participants analysed	/
Outcome	For the primary outcome, a result for each group and the estimated effect size and its precision	/
Harms	Important adverse events or side-effects	10-12
Conclusions	General interpretation of the results	/
Trial registration	Registration number and name of trial register, if applicable	1
Funding	Source of funding	/
