# Supplementary material for: Efficacy and Safety of Ginkgo Biloba Pills for Coronary Heart Disease with Impaired Glucose Regulation: Study Protocol for a Series of N-of-1 Randomized, Double-Blind, Placebo-Controlled Trials
Source: Evid Based Complement Alternat Med. 2018 Oct 14;2018:7571629. doi: 10.1155/2018/7571629 (PMC6204161; doi:10.1155/2018/7571629)
Supplement: Supplementary 3 — Additional file 3: Case report form. [file 7571629.f3.pdf]

A single-case randomized controlled trial of Ginkgo Biloba pills in the treatment of coronary heart disease with impaired glucose regulation

Case Report Form

Research Center: Xiyuan Hospital, China Academy of Chinese Medical Sciences

Subject initials: |___|___|___|___|

Subject screening number: |___|______|___|

Drug number: |___|___|___|

Sponsor: Wanbangde Pharmaceutical Group Co., Ltd.

Please read the following instructions carefully before filling out the form.

Fill in the case report form

1. Fill in the CRF for all eligible cases.

2. Always use a black or blue-black pen to fill in the records..The writing should be legible and easy to read;you cannot use a ballpoint pen or pencil.

3. The CRF must be filled in accurately and clearly.It should not be altered at will. If the error is corrected,it should be marked with a horizontal line and the name of the modifier and the time of modification.Exampl:~~99.6~~ 90.6 LGW 00 02 12.

4. The patient's name is abbreviated in four digits. The two characters are filled in the first two abbreviations of the two-character pinyin; the three-character name is filled with the three-character abbreviation and the third-character second abbreviation; the four-character name is filled in the first abbreviation of each word. Example: Zhang Hong | Z | H | H | O|; Li Shuming | L | S | M | I |; Ouyang Xiaohui | O | Y | X | H |.

5. Please fill in the numbers in the open spaces and fill in a number for each box. If the number of digits is not enough, please add zeros in the space before or after. For example: weight 58kg, filled in the space: | 5 | 8 |.| 0 | kg; blood pressure 120/80 mgHg, filled in the space: | 1 | 2 | 0 |/| 0 | 8 | 0 |mmHg

6. All selected items should be marked with × in the matching “□”, such as: ☒.

7. The record format of the date in the CRF is in the international ISO 8601 date format (yyyymmdd); all time records are in 24-hour format (for example, 15:00 in the afternoon is 15:00 instead of 03:00pm), and the recording in the middle of the night is 00:00 (Not 24:00) and as a new day.

8. Please answer all the questions in the CRF and don't leave a blank in the question. If “not done” fills in “ND”, “not applicable” fills in “NA”, “cannot get” fills in “UA”, “unknown” fills in “UK”, etc.,and other cases are described in words.Pages without any records should also complete the header section and draw a diagonal slash on the page.

9.Do not change the format of the CRF.If there is no corresponding location in the discovery form or the information is not enough to record the information that the person wants to record, please record the information in the blank “attachment” on page 24 and sign it on the page.

10. At the appropriate location on each page of the form, ask the investigator to sign and complete the date of signature.

11. The adverse event record form should be filled in truthfully during the test. Record the time, severity, duration, measures taken, and outcomes of adverse events.If serious adverse events occur (including hospitalization, prolonged hospitalization, disability, work ability, life-threatening or death, congenital malformation, etc.), the ethics committee of the responsible unit must be notified immediately, contract research organization.

Baseline -7~0Day	Drug number |___|___|___|	Patient initials |___|___|___|___|	Date of visit 201|__|Year|__|__|Month|__|__|Day

Informed consent

Informed consent date： |___|___|___|___|Year|___|___|Month|___|___|Day

Demographic data

Age： |___|___|___|___|Year|___|___|Month|___|___|Day	Sex： □1Male □2Female
Nationality： □1 Han nationality □2 Other： __________	Nature of work: □1 physical strength □2 non-physical strength
Height： |___|___|___| cm	Body Weight： |___|___|.|___| kg

Diagnosis

**Western medicine diagnosis**
Western medicine diagnosis： □Coronary heart disease
Course： |___|___|___|Month

History of treatment for coronary heart disease: Has the patient been treated for the disease?
□0 no □1 is → please fill in the details

Drug name	Dosage	Start date	End date

Past history: Does the patient have other diseases and medications at the moment:
□0 no □1 is → please fill in the details

Diagnosis	Drug/Treatment name	Dosage	Start date	End date	Still using
			|__|__|__|__|Year|__|__|Month|__|__|Day	|__|__|__|__|Year|__|__|Month|__|__|Day	□1
			|__|__|__|__|Year|__|__|Month|__|__|Day	|__|__|__|__|Year|__|__|Month|__|__|Day	□1
			|__|__|__|__|Year|__|__|Month|__|__|Day	|__|__|__|__|Year|__|__|Month|__|__|Day	□1

History of allergies: Does the patient have a history of dication/food/environmental allergies?

□0 no □1 is → please fill in the details ____

Baseline -7~0Day	Drug number |___|___|___|	Patient initials |___|___|___|___|	Date of visit 201|__|Year|__|__|Month|__|__|Day

Vital signs

Body temperature (℃)	Breathe (Times/minute)	Heart rate (Times/minute)	Blood pressure (mmHg)
|___|___|.|___|	|___|___|	|___|___|___|	|___|___|___|/|___|___|___|

Seattle Angina Questionnaire(SAQ) Score *：\|___\|___\|___\|

1. In the past 4 weeks, the following restrictions have been caused by chest pain, chest tightness and angina:

	Severely restricted	Moderately restricted	Mildly restricted	Slightly restricted	Unrestricted	Limited for other reasons
Dress yourself	□1	□2	□3	□4	□5	□6
Indoor walk	□1	□2	□3	□4	□5	□6
Take a shower	□1	□2	□3	□4	□5	□6
Climbing or stairway (three floors, non-stop)	□1	□2	□3	□4	□5	□6
Outdoor activities or pick up	□1	□2	□3	□4	□5	□6
Walk lightly (one kilometer)	□1	□2	□3	□4	□5	□6
Jogging (one kilometer)	□1	□2	□3	□4	□5	□6
Lift or move heavy objects	□1	□2	□3	□4	□5	□6
Strenuous exercise (such as swimming or playing)	□1	□2	□3	□4	□5	□6

2. Compared with 4 weeks ago, when doing the maximum intensity of activity, chest pain, chest tightness and angina pectoris:
□1 obviously increase □2 Slight increase □3 the same □4 Minor reduction □5 obviously decrease

3. The average number of episodes of chest pain, chest tightness, and angina in the past 4 weeks:

□1 ≥four times/day □2 0ne-three times/day □3≥three times/week □4 one-two times/week

□5 < 1 time/week □6 no episode

4. The average number of nitro drugs (such as nitroglycerin) in chest pain, chest tightness, and angina pectoris over the past 4 weeks:

□1 ≥four times/day □2 0ne-three times/day □3≥three times/week □4 one-two times/week

□5 < 1 time/week □6 no episode

5. Due to chest pain, chest tightness and angina pectoris, follow the doctor's advice:

□1 Serious □2 Moderate □3 Minor □4 Very little □5 No □6 Doctor did not administer

6. Satisfaction with various measures for treating chest pain, chest tightness and angina:

□1 Dissatisfied □2 Most dissatisfied □3 Partially satisfied □4 Most satisfied □5 Highly satisfied

Baseline	Drug number	Patient initials	Date of visit
-7~0Day	|___|___|___|	|___|___|___|___|	201|__|Year|__|__|Month|__|__|Day

7. Satisfaction with the doctor's explanation for chest pain, chest tightness and angina:

□$_1$ Dissatisfied □$_2$ Most dissatisfied □$_3$ Partially satisfied □$_4$ Most satisfied □$_5$ Highly satisfied

8. Overall, satisfaction with the current treatment of chest pain, chest tightness and angina:

□$_1$ Dissatisfied □$_2$ Most dissatisfied □$_3$ Partially satisfied □$_4$ Most satisfied □$_5$ Highly satisfied

9. In the past 4 weeks, chest pain, chest tightness and angina have affected the level of fun in life:

□$_1$ Dissatisfied □$_2$ Most dissatisfied □$_3$ Partially satisfied □$_4$ Most satisfied □$_5$ Highly satisfied

10. If you have chest pain, chest tightness and angina in your future life, how would you feel?

□$_1$ Dissatisfied □$_2$ Most dissatisfied □$_3$Partially satisfied □$_4$ Most satisfied □$_5$ Highly satisfied

11. Concerns about heart attack and sudden death:

□$_1$ Always worried □$_2$ Frequently worried □$_3$ Sometimes worried □$_4$ Very little worried □$_5$ Never worry

* The Seattle Angina Scale is divided into 5 items and 19 items: Physical activity limitation (PL, question l), angina stable state (AS, question 2), angina pectoris (AF, question 3-4), treatment satisfaction (TS), question 5-8), disease cognition (DP, question 9-11). After scoring the 19 items of the 5 major items one by one, each score is forwarded.The standard score = (actual score - the lowest score in this aspect) / (the highest score in this aspect - the lowest score in this aspect) ✖100, the higher the score, the better the quality of life and the functional state of the patient.

Angina pectoris symptom score Score *: |___|___|___|

Number of angina attacks	□$_0$ No	□$_2$ 2-6 episodes per week	□$_4$1-3 times a day	□$_6$ more than 4 episodes per day
Angina duration	□$_0$ No	□$_2$ Each time the pain lasts less than or equal to 5 minutes	□$_4$ Each time the pain lasts longer than 5 minutes and less than 10	□$_6$ Each time the pain lasts for 10 minutes or more
Angina pain level	□$_0$ No	□$_2$ Physical activity that is heavier than daily activities causes angina and daily activities are asymptomatic. Activities that can cause angina, such as trotting on the ground, fast or holding heavy objects on the third floor, up steep slopes, etc.	□$_4$ Daily physical activity causes angina and daily activities are slightly limited. Activities that can cause angina, such as walking 3-4 stations (3-4 miles) at normal speed under normal conditions, on the third floor, uphill, etc.	□$_6$ Lighter physical activity than daily activities causes angina and daily activities are significantly limited. Activities that can cause angina, such as walking 1-2 stations (1-2 miles) at normal speed under normal conditions, on the second floor, gentle slope, etc.
Nitroglycerin dosage	□$_0$ No	□$_2$ 1-4 tablets per week	□$_4$ 5-9tablets per week	□$_6$ Take more than 10 tablets a week

Baseline -7~0Day	Drug number |___|___|___|	Patient initials |___|___|___|___|	Date of visit 201|__|Year|__|__|Month|__|__|Day

Laboratory inspection

Indicator	Measured Value	Unit	Clinical significance * 1 2 3 4	Anomalous cause explanation
**Blood routine**		**Report date:** 201|___|Year|___|___|Month|___|___|Day		
HGB		g/L	□1 □2 □3 □4	
RBC		$\times 10^{12}$L	□1 □2 □3 □4	
WBC		$\times 10^{9}$/L	□1 □2 □3 □4	
PLT		$\times 10^{9}$/L	□1 □2 □3 □4	
**Urine routine**		**Report date:** 201|___|Year|___|___|Month|___|___|Day		
Urine PH			□1 □2 □3 □4	
PRO		—	□1 □2 □3 □4	
ERY		/HPF	□**1** □**2** □**3** □**4**	
White blood cells（LEU）		/HPF	□**1** □**2** □**3** □**4**	
**Liver and kidney function**		**Report date:** 201|___|Year|___|___|Month|___|___|Day		
ALT		U/L	□1 □2 □3 □4	
AST		U/L	□1 □2 □3 □4	
ALP		U/L	□1 □2 □3 □4	
TBIL		μmol/L	□1 □2 □3 □4	
γ-GT		U/L	□1 □2 □3 □4	
TBA		μmol/L	□1 □2 □3 □4	
BUN		mmol/L	□1 □2 □3 □4	
Cr		μmol/L	□1 □2 □3 □4	
**blood sugar**		**Report date:** 201|___|Year|___|___|Month|___|___|Day		
FPG		mmol/L	□1 □2 □3 □4	
2hPG		mmol/L	□1 □2 □3 □4	
HbA1c		mg/dl	□1 □2 □3 □4	
Fasting insulin		μU/ml	□1 □2 □3 □4	

Baseline -7~0Day	Drug number |___|___|___|	Patient initials |___|___|___|___|	Date of visit 201|__|Year|__|__|Month|__|__|Day

**Blood lipid**		**Report date:** 201|___|Year|___|___|Month|___|___|Day		
HDL-C		mmol/L	□1 □2 □3 □4	
LDL-C		mmol/L	□1 □2 □3 □4	
TG		mmol/L	□1 □2 □3 □4	
CH		mmol/L	□1 □2 □3 □4	
**C-reactive protein**		**Report date:** 201|___|Year|___|___|Month|___|___|Day		
CRP		㎎/L	□1 □2 □3 □4	
**Electrocardiogram**		**Report date:** 201|___|Year|___|___|Month|___|___|Day		
ECG		—	□1 □2 □3 □4	
**Urine pregnancy**		**Report date:** 201|___|Year|___|___|Month|___|___|Day		
Urine pregnancy		—	□1 Not applicable (male,post-sterilization or menopause for more than 1 year women)<br>□2 Negative<br>□3 Positive<br>(Can't enter the test)<br>□4 未查	

* Clinical significance judgment: (1) normal (2) abnormal but no clinical significance (3) abnormal and clinical significance (4) not investigated.

Baseline -7~0Day	Drug number	Patient initials	Date of visit
	|___|___|___|	|___|___|___|___|	201|__|Year|__|__|Month|__|__|Day

Inclusion criteria: The following options are all "Yes" to be selected.

1. Meet the diagnostic criteria for coronary heart disease Western medicine (any one can be):<br>a) have a clear history of old myocardial infarction, or have a history of PCI, or have a history of bypass (at least 3 months);<br>b) Coronary angiography or coronary CTA results suggest that at least one coronary artery is narrowed and the degree of lumen stenosis is ≥50%;	□$_1$ Yes	□$_0$ No
2. In line with the criteria for stable angina, and the number of episodes of angina pectoris ≥ 2 times per week;	□$_1$ Yes	□$_0$ No
3. Comply with the 25 diagnostic criteria of blood stasis syndrome of coronary heart disease（CHD）;	□$_1$ Yes	□$_0$ No
4. Comply with the 2016 Diabetes Association (ADA) published criteria for impaired diagnosis of glucose regulation;	□$_1$ Yes	□$_0$ NO
5.Aged between 18 and 75 years，no gender；	□$_1$ Yes	□$_0$ No
6. Participants voluntarily participated in this study, signed informed consent and had good compliance.	□$_1$ Yes	□$_0$ No

Baseline -7~0Day	Drug number |___|___|___|	Patient initials |___|___|___|___|	Date of visit 201|__|Year|__|__|Month|__|__|Day

Exclusion criteria: The following options are all "No" to be selected.

1.With congenital or 5 rheumatic heart disease or severe cardiopulmonary insufficiency (grade 3 and 4 of 6 cardiac function),or uncontrolled severe arrhythmias(includingventricular7 tachycardia,supraventricular tachycardia),or not controlled hypertension (systolic 8 blood pressure ≥160 mmHg or diastolic blood pressure ≥100 mmHg);	□$_1$ Yes	□$_0$ No
2. With cerebrovascular disease, or with severe liver and kidney dysfunction, or with 10 endocrine, urinary, blood system and other serious primary diseases;	□$_1$ Yes	□$_0$ No
3. Within 4 weeks, there was history of major organ surgery such as head, chest or abdomen or bleeding tendency;	□$_1$ Yes	□$_0$ No
4. Those who have taken hypoglycaemic agents or glucocorticoids, thiazide diuretics and other drugs that affect blood sugar levels within 3 months;	□$_1$ Yes	□$_0$ No
5. People with diseases affecting blood glucose metabolism, such as thyroid glands and adrenal diseases,or those with previous history of the aforementioned diseases;	□$_1$ Yes	□$_0$ No
6. Allergies or persons allergic to known ingredients of the study drug;	□$_1$ Yes	□$_0$ No
7.Pregnancy and lactation women or those with a pregnancy plan;	□$_1$ Yes	□$_0$ No
8. Subjects who participated in other clinical trials in the last 3 months;	□$_1$ Yes	□$_0$ No
9. Researchers consider that subjects should not participate in clinical trials.	□$_1$ Yes	□$_0$ No

Screening results: Does the patient enter the trial?

□$_1$Yes→Drug number: |___|___|___|

Drug delivery time: 201|___|Year|___|___|Month|___|___|Day

Number of medicines: 900 pills (10 bottles)

□$_0$No→Please indicate the reason and fill in the screening selection form: ________________________

Researcher's signature: ____________________**Date**: 201|___|Year|___|___|Month|___|___|Day

Visit1 8Week±4Day	Drug number	Patient initials	Date of visit
	|__|__|__|	|__|__|__|__|	201|__|Year|__|__|Month|__|__|Day

Vital signs

Body temperature (℃)	Breathe (Times/minute)	Heart rate (Times/minute)	Blood pressure (mmHg)
|__|__|.|__|	|__|__|	|__|__|__|	|__|__|__|/|__|__|__|

Seattle Angina Questionnaire(SAQ) Score *：\|__\|__\|__\|

1. In the past 4 weeks, the following restrictions have been caused by chest pain, chest tightness and angina:

	Severely restricted	Moderately restricted	Mildly restricted	Slightly restricted	Unrestricted	Limited for other reasons
Dress yourself	□1	□2	□3	□4	□5	□6
Indoor walk	□1	□2	□3	□4	□5	□6
Take a shower	□1	□2	□3	□4	□5	□6
Climbing or stairway (three floors, non-stop)	□1	□2	□3	□4	□5	□6
Outdoor activities or pick up	□1	□2	□3	□4	□5	□6
Walk lightly (one kilometer)	□1	□2	□3	□4	□5	□6
Jogging (one kilometer)	□1	□2	□3	□4	□5	□6
Lift or move heavy objects	□1	□2	□3	□4	□5	□6
Strenuous exercise (such as swimming or playing)	□1	□2	□3	□4	□5	□6

2. Compared with 4 weeks ago, when doing the maximum intensity of activity, chest pain, chest tightness and angina pectoris:
□1 obviously increase □2 Slight increase □3 the same □4 Minor reduction □5 obviously decrease

3. The average number of episodes of chest pain, chest tightness, and angina in the past 4 weeks:

□1 ≥four times/day □2 0ne-three times/day □3≥three times/week □4 one-two times/week

□5 < 1 time/week □6 no episode

4. The average number of nitro drugs (such as nitroglycerin) in chest pain, chest tightness, and angina pectoris over the past 4 weeks:

□1 ≥four times/day □2 0ne-three times/day □3≥three times/week □4 one-two times/week

□5 < 1 time/week □6 no episode

5. Due to chest pain, chest tightness and angina pectoris, follow the doctor's advice:

□1 Serious □2 Moderate □3 Minor □4 Very little □5 No □6 Doctor did not administer

Visit1 8Week±4Day	Drug number	Patient initials	Date of visit
	|___|___|___|	|___|___|___|___|	201|__|Year|__|__|Month|__|__|Day

6. Satisfaction with various measures for treating chest pain, chest tightness and angina:

□1 Dissatisfied □2 Most dissatisfied □3 Partially satisfied □4 Most satisfied □5 Highly satisfied

7. Satisfaction with the doctor's explanation for chest pain, chest tightness and angina:

□1 Dissatisfied □2 Most dissatisfied □3 Partially satisfied □4 Most satisfied □5 Highly satisfied

8. Overall, satisfaction with the current treatment of chest pain, chest tightness and angina:

□1 Dissatisfied □2 Most dissatisfied □3 Partially satisfied □4 Most satisfied □5 Highly satisfied

9. In the past 4 weeks, chest pain, chest tightness and angina have affected the level of fun in life:

□1 Dissatisfied □2 Most dissatisfied □3 Partially satisfied □4 Most satisfied □5 Highly satisfied

10. If you have chest pain, chest tightness and angina in your future life, how would you feel?

□1 Dissatisfied □2 Most dissatisfied □3Partially satisfied □4 Most satisfied □5 Highly satisfied

11. Concerns about heart attack and sudden death:

□1 Always worried □2 Frequently worried □3 Sometimes worried □4 Very little worried □5 Never worry

* The Seattle Angina Scale is divided into 5 items and 19 items: Physical activity limitation (PL, question l), angina stable state (AS, question 2), angina pectoris (AF, question 3-4), treatment satisfaction (TS), question 5-8), disease cognition (DP, question 9-11). After scoring the 19 items of the 5 major items one by one, each score is forwarded.The standard score = (actual score - the lowest score in this aspect) / (the highest score in this aspect - the lowest score in this aspect) ✖100, the higher the score, the better the quality of life and the functional state of the patient.

Angina pectoris symptom score Score *: |___|___|___|

Number of angina attacks	□0 No	□2 2-6 episodes per week	□4 1-3 times a day	□6 more than 4 episodes per day
Angina duration	□0 No	□2 Each time the pain lasts less than or equal to 5 minutes	□4 Each time the pain lasts longer than 5 minutes and less than 10	□6 Each time the pain lasts for 10 minutes or more
Angina pain level	□0 No	□2 Physical activity that is heavier than daily activities causes angina and daily activities are asymptomatic. Activities that can cause angina, such as trotting on the ground, fast or holding heavy objects on the third floor, up steep slopes, etc.	□4 Daily physical activity causes angina and daily activities are slightly limited. Activities that can cause angina, such as walking 3-4 stations (3-4 miles) at normal speed under normal conditions, on the third floor, uphill, etc.	□6 Lighter physical activity than daily activities causes angina and daily activities are significantly limited. Activities that can cause angina, such as walking 1-2 stations (1-2 miles) at normal speed under normal conditions, on the second floor, gentle slope, etc.
Nitroglycerin dosage	□0 No	□2 1-4 tablets per week	□4 5-9tablets per week	□6 Take more than 10 tablets a week

Visit1<br>8Week±4Day	Drug number	Patient initials	Date of visit
	|___|___|___|	|___|___|___|___|	201|__|Year|__|__|Month|__|__|Day

Laboratory inspection

Indicator	Measured Value	Unit	Clinical significance * 1 2 3 4	Anomalous cause explanation
**Blood routine**		**Report date:** 201|___|Year|___|___|Month|___|___|Day		
HGB		g/L	□1 □2 □3 □4	
RBC		$\times 10^{12}$L	□1 □2 □3 □4	
WBC		$\times 10^{9}$/L	□1 □2 □3 □4	
PLT		$\times 10^{9}$/L	□1 □2 □3 □4	
**Urine routine**		**Report date:** 201|___|Year|___|___|Month|___|___|Day		
Urine PH			□1 □2 □3 □4	
PRO		—	□1 □2 □3 □4	
ERY		/HPF	**□1 □2 □3 □4**	
White blood cells (LEU)		/HPF	**□1 □2 □3 □4**	
**Liver and kidney function**		**Report date:** 201|___|Year|___|___|Month|___|___|Day		
ALT		U/L	□1 □2 □3 □4	
AST		U/L	□1 □2 □3 □4	
ALP		U/L	□1 □2 □3 □4	
TBIL		µmol/L	□1 □2 □3 □4	
γ-GT		U/L	□1 □2 □3 □4	
TBA		µmol/L	□1 □2 □3 □4	
BUN		mmol/L	□1 □2 □3 □4	
Cr		µmol/L	□1 □2 □3 □4	
**blood sugar**		**Report date:** 201|___|Year|___|___|Month|___|___|Day		
FPG		mmol/L	□1 □2 □3 □4	
2hPG		mmol/L	□1 □2 □3 □4	
HbA1c		mg/dl	□1 □2 □3 □4	
Fasting insulin		µU/ml	□1 □2 □3 □4	

Visit1 8Week±4Day	Drug number |___|___|___|	Patient initials |___|___|___|___|	Date of visit 201|__|Year|__|__|Month|__|__|Day

**Blood lipid**		**Report date:** 201|___|Year|___|___|Month|___|___|Day		
HDL-C		mmol/L	□1 □2 □3 □4	
LDL-C		mmol/L	□1 □2 □3 □4	
TG		mmol/L	□1 □2 □3 □4	
CH		mmol/L	□1 □2 □3 □4	
**C-reactive protein**		**Report date:** 201|___|Year|___|___|Month|___|___|Day		
CRP		mg/L	□1 □2 □3 □4	
**Electrocardiogram**		**Report date:** 201|___|Year|___|___|Month|___|___|Day		
ECG		—	□1 □2 □3 □4	

* Clinical significance judgment: (1) normal (2) abnormal but no clinical significance (3) abnormal and clinical significance (4) not investigated.

Is the test drug returned?

□0No→Unreturned reason: □2 Take all □3 Lost □4 Other___________
□1Yes→ Ginkgo biloba drop pills |___|___|___| pills

Is there a combined medication?

□0No
□1Yes→Please fill out the "Combined Medication Record Form" on page 47.

Are there any adverse events?

□0No
□1Yes→Please fill out the "Adverse Event Record Form" on page 48 in detail.

Is there an unscheduled laboratory check?

□0No
□1Yes→Record the results to "Unplanned Laboratory Inspection Results" on page 49

Researcher's signature: ____________________ **Date**: 201\|___\|Year\|___\|___\|Month\|___\|___\|Day

Visit2<br>10Week±4Day	Drug number |__|__|__|	Patient initials |__|__|__|__|	Date of visit<br>201|__|Year|__|__|Month|__|__|Day

Seattle Angina Questionnaire(SAQ) Score *：|__|__|__|

1.In the past 4 weeks, the following restrictions have been caused by chest pain, chest tightness and angina:

	Severely restricted	Moderately restricted	Mildly restricted	Slightly restricted	Unrestricted	Limited for other reasons
Dress yourself	□1	□2	□3	□4	□5	□6
Indoor walk	□1	□2	□3	□4	□5	□6
Take a shower	□1	□2	□3	□4	□5	□6
Climbing or stairway (three floors, non-stop)	□1	□2	□3	□4	□5	□6
Outdoor activities or pick up	□1	□2	□3	□4	□5	□6
Walk lightly (one kilometer)	□1	□2	□3	□4	□5	□6
Jogging (one kilometer)	□1	□2	□3	□4	□5	□6
Lift or move heavy objects	□1	□2	□3	□4	□5	□6
Strenuous exercise (such as swimming or playing)	□1	□2	□3	□4	□5	□6

2. Compared with 4 weeks ago, when doing the maximum intensity of activity, chest pain, chest tightness and angina pectoris:
□1 obviously increase □2 Slight increase □3 the same □4 Minor reduction □5 obviously decrease

3. The average number of episodes of chest pain, chest tightness, and angina in the past 4 weeks:

□1 ≥four times/day □2 0ne-three times/day □3≥three times/week □4 one-two times/week

□5 < 1 time/week □6 no episode

4. The average number of nitro drugs (such as nitroglycerin) in chest pain, chest tightness, and angina pectoris over the past 4 weeks:

□1 ≥four times/day □2 0ne-three times/day □3≥three times/week □4 one-two times/week

□5 < 1 time/week □6 no episode

5. Due to chest pain, chest tightness and angina pectoris, follow the doctor's advice:

□1 Serious □2 Moderate □3 Minor □4 Very little □5 No □6 Doctor did not administer

6. Satisfaction with various measures for treating chest pain, chest tightness and angina:

□1 Dissatisfied □2 Most dissatisfied □3 Partially satisfied □4 Most satisfied □5 Highly satisfied

7. Satisfaction with the doctor's explanation for chest pain, chest tightness and angina:

□1 Dissatisfied □2 Most dissatisfied □3 Partially satisfied □4 Most satisfied □5 Highly satisfied

8. Overall, satisfaction with the current treatment of chest pain, chest tightness and angina:

□1 Dissatisfied □2 Most dissatisfied □3 Partially satisfied □4 Most satisfied □5 Highly satisfied

9. In the past 4 weeks, chest pain, chest tightness and angina have affected the level of fun in life:

□$_1$ Dissatisfied □$_2$ Most dissatisfied □$_3$ Partially satisfied □$_4$ Most satisfied □$_5$ Highly satisfied

10. If you have chest pain, chest tightness and angina in your future life, how would you feel?

□$_1$ Dissatisfied □$_2$ Most dissatisfied □$_3$Partially satisfied □$_4$ Most satisfied □$_5$ Highly satisfied

11. Concerns about heart attack and sudden death:

□$_1$ Always worried □$_2$ Frequently worried □$_3$ Sometimes worried □$_4$ Very little worried □$_5$ Never worry

* The Seattle Angina Scale is divided into 5 items and 19 items: Physical activity limitation (PL, question l), angina stable state (AS, question 2), angina pectoris (AF, question 3-4), treatment satisfaction (TS), question 5-8), disease cognition (DP, question 9-11). After scoring the 19 items of the 5 major items one by one, each score is forwarded.The standard score = (actual score - the lowest score in this aspect) / (the highest score in this aspect - the lowest score in this aspect) ✖100, the higher the score, the better the quality of life and the functional state of the patient.

Angina pectoris symptom score Score *: |___|___|___|

Number of angina attacks	□$_0$ No	□$_2$ 2-6 episodes per week	□$_4$1-3 times a day	□$_6$ more than 4 episodes per day
Angina duration	□$_0$ No	□$_2$ Each time the pain lasts less than or equal to 5 minutes	□$_4$ Each time the pain lasts longer than 5 minutes and less than 10	□$_6$ Each time the pain lasts for 10 minutes or more
Angina pain level	□$_0$ No	□$_2$ Physical activity that is heavier than daily activities causes angina and daily activities are asymptomatic. Activities that can cause angina, such as trotting on the ground, fast or holding heavy objects on the third floor, up steep slopes, etc.	□$_4$ Daily physical activity causes angina and daily activities are slightly limited. Activities that can cause angina, such as walking 3-4 stations (3-4 miles) at normal speed under normal conditions, on the third floor, uphill, etc.	□$_6$ Lighter physical activity than daily activities causes angina and daily activities are significantly limited. Activities that can cause angina, such as walking 1-2 stations (1-2 miles) at normal speed under normal conditions, on the second floor, gentle slope, etc.
Nitroglycerin dosage	□$_0$ No	□$_2$ 1-4 tablets per week	□$_4$ 5-9tablets per week	□$_6$ Take more than 10 tablets a week

Visit2<br>10Week±4Day	Drug number<br>|___|___|___|	Patient initials<br>|___|___|___|___|	Date of visit<br>201|__|Year|__|__|Month|__|__|Day

Laboratory inspection

Indicator	Measured Value	Unit	Clinical significance *<br>1 2 3 4	Anomalous cause explanation
**Blood sugar**		**Report date:** 201|___|Year|___|___|Month|___|___|Day		
FPG		mmol/L	□1 □2 □3 □4	
2hPG		mmol/L	□1 □2 □3 □4	
HbA1c		mg/dl	□1 □2 □3 □4	
Fasting insulin		μU/ml	□1 □2 □3 □4	
**Blood lipid**		**Report date:** 201|___|Year|___|___|Month|___|___|Day		
HDL-C		mmol/L	□1 □2 □3 □4	
LDL-C		mmol/L	□1 □2 □3 □4	
TG		mmol/L	□1 □2 □3 □4	
CH		mmol/L	□1 □2 □3 □4	
**C-reactive protein**		**Report date:** 201|___|Year|___|___|Month|___|___|Day		
CRP		mg/L	□1 □2 □3 □4	

* Clinical significance judgment: (1) normal (2) abnormal but no clinical significance (3) abnormal and clinical significance (4) not investigated.

Is there a combined medication?

□0No

□1Yes→Please fill out the “Combined Medication Record Form” on page 47.

Are there any adverse events?

□0No

□1Yes→Please fill out the “Adverse Event Record Form” on page 48 in detail.

Is there an unscheduled laboratory check?

□0No

□1Yes→Record the results to “Unplanned Laboratory Inspection Results” on page 49

Release of test drug: Ginkgo biloba drop pills 900 pills (10 bottles)

Researcher's signature: ____________________**Date**: 201|___|Year|___|___|Month|___|___|Day

Visit3<br>18Week±4day	Drug number<br>|___|___|___|	Patient initials<br>|___|___|___|___|	Date of visit<br>201|__|Year|__|__|Month|__|__|Day

Seattle Angina Questionnaire(SAQ) Score *：|___|___|___|

1. In the past 4 weeks, the following restrictions have been caused by chest pain, chest tightness and angina:

	Severely restricted	Moderately restricted	Mildly restricted	Slightly restricted	Unrestricted	Limited for other reasons
Dress yourself	□1	□2	□3	□4	□5	□6
Indoor walk	□1	□2	□3	□4	□5	□6
Take a shower	□1	□2	□3	□4	□5	□6
Climbing or stairway (three floors, non-stop)	□1	□2	□3	□4	□5	□6
Outdoor activities or pick up	□1	□2	□3	□4	□5	□6
Walk lightly (one kilometer)	□1	□2	□3	□4	□5	□6
Jogging (one kilometer)	□1	□2	□3	□4	□5	□6
Lift or move heavy objects	□1	□2	□3	□4	□5	□6
Strenuous exercise (such as swimming or playing)	□1	□2	□3	□4	□5	□6

2. Compared with 4 weeks ago, when doing the maximum intensity of activity, chest pain, chest tightness and angina pectoris:
□1 obviously increase □2 Slight increase □3 the same □4 Minor reduction □5 obviously decrease

3. The average number of episodes of chest pain, chest tightness, and angina in the past 4 weeks:

□1 ≥four times/day □2 0ne-three times/day □3≥three times/week □4 one-two times/week

□5 < 1 time/week □6 no episode

4. The average number of nitro drugs (such as nitroglycerin) in chest pain, chest tightness, and angina pectoris over the past 4 weeks:

□1 ≥four times/day □2 0ne-three times/day □3≥three times/week □4 one-two times/week

□5 < 1 time/week □6 no episode

5. Due to chest pain, chest tightness and angina pectoris, follow the doctor's advice:

□1 Serious □2 Moderate □3 Minor □4 Very little □5 No □6 Doctor did not administer

6. Satisfaction with various measures for treating chest pain, chest tightness and angina:

□1 Dissatisfied □2 Most dissatisfied □3 Partially satisfied □4 Most satisfied □5 Highly satisfied

7. Satisfaction with the doctor's explanation for chest pain, chest tightness and angina:

□1 Dissatisfied □2 Most dissatisfied □3 Partially satisfied □4 Most satisfied □5 Highly satisfied

8. Overall, satisfaction with the current treatment of chest pain, chest tightness and angina:

□1 Dissatisfied □2 Most dissatisfied □3 Partially satisfied □4 Most satisfied □5 Highly satisfied

Visit3 18Week±4day	Drug number	Patient initials	Date of visit
	|___|___|___|	|___|___|___|___|	201|__|Year|__|__|Month|__|__|Day

9. In the past 4 weeks, chest pain, chest tightness and angina have affected the level of fun in life:

□1 Dissatisfied □2 Most dissatisfied □3 Partially satisfied □4 Most satisfied □5 Highly satisfied

10. If you have chest pain, chest tightness and angina in your future life, how would you feel?

□1 Dissatisfied □2 Most dissatisfied □3Partially satisfied □4 Most satisfied □5 Highly satisfied

11. Concerns about heart attack and sudden death:

□1 Always worried □2 Frequently worried □3 Sometimes worried □4 Very little worried □5 Never worry

* The Seattle Angina Scale is divided into 5 items and 19 items: Physical activity limitation (PL, question l), angina stable state (AS, question 2), angina pectoris (AF, question 3-4), treatment satisfaction (TS), question 5-8), disease cognition (DP, question 9-11). After scoring the 19 items of the 5 major items one by one, each score is forwarded.The standard score = (actual score - the lowest score in this aspect) / (the highest score in this aspect - the lowest score in this aspect) ✖100, the higher the score, the better the quality of life and the functional state of the patient.

Angina pectoris symptom score Score *: |___|___|___|

Number of angina attacks	□0 No	□2 2-6 episodes per week	□41-3 times a day	□6 more than 4 episodes per day
Angina duration	□0 No	□2 Each time the pain lasts less than or equal to 5 minutes	□4 Each time the pain lasts longer than 5 minutes and less than 10	□6 Each time the pain lasts for 10 minutes or more
Angina pain level	□0 No	□2 Physical activity that is heavier than daily activities causes angina and daily activities are asymptomatic. Activities that can cause angina, such as trotting on the ground, fast or holding heavy objects on the third floor, up steep slopes, etc.	□4 Daily physical activity causes angina and daily activities are slightly limited. Activities that can cause angina, such as walking 3-4 stations (3-4 miles) at normal speed under normal conditions, on the third floor, uphill, etc.	□6 Lighter physical activity than daily activities causes angina and daily activities are significantly limited. Activities that can cause angina, such as walking 1-2 stations (1-2 miles) at normal speed under normal conditions, on the second floor, gentle slope, etc.
Nitroglycerin dosage	□0 No	□2 1-4 tablets per week	□4 5-9tablets per week	□6 Take more than 10 tablets a week

Visit3<br>18Week±4day	Drug number |__|__|__|	Patient initials |__|__|__|__|	Date of visit<br>201|__|Year|__|__|Month|__|__|Day

Laboratory inspection

Indicator	Measured Value	Unit	Clinical significance *<br>1 2 3 4	Anomalous cause explanation
**Blood sugar**		**Report date:** 201|__|Year|__|__|Month|__|__|Day		
FPG		mmol/L	□1 □2 □3 □4	
2hPG		mmol/L	□1 □2 □3 □4	
HbA1c		mg/dl	□1 □2 □3 □4	
Fasting insulin		μU/ml	□1 □2 □3 □4	
**Blood lipid**		**Report date:** 201|__|Year|__|__|Month|__|__|Day		
HDL-C		mmol/L	□1 □2 □3 □4	
LDL-C		mmol/L	□1 □2 □3 □4	
TG		mmol/L	□1 □2 □3 □4	
CH		mmol/L	□1 □2 □3 □4	
**C-reactive protein**		**Report date:** 201|__|Year|__|__|Month|__|__|Day		
CRP		mg/L	□1 □2 □3 □4	

* Clinical significance judgment: (1) normal (2) abnormal but no clinical significance (3) abnormal and clinical significance (4) not investigated.

Visit3 18Week±4day	Drug number |___|___|___|	Patient initials |___|___|___|___|	Date of visit 201|__|Year|__|__|Month|__|__|Day

Is the test drug returned?

□$_0$No→Unreturned reason： □$_2$ Take all □$_3$ Lost □$_4$ Other___________
□$_1$Yes→ Ginkgo biloba drop pills |___|___|___| pills

Is there a combined medication?

□$_0$No
□$_1$Yes→Please fill out the "Combined Medication Record Form" on page 47.

Are there any adverse events?

□$_0$No
□$_1$Yes→Please fill out the "Adverse Event Record Form" on page 48 in detail.

Is there an unscheduled laboratory check?

□$_0$No
□$_1$Yes→Record the results to "Unplanned Laboratory Inspection Results" on page 49

Researcher's signature: ____________________**Date**: 201|___|Year|___|___|Month|___|___|Day

Visit4<br>20Week±4Day	Drug number<br>|___|___|___|	Patient initials<br>|___|___|___|___|	Date of visit<br>201|__|Year|__|__|Month|__|__|Day

Seattle Angina Questionnaire(SAQ) Score *：|___|___|___|

1. In the past 4 weeks, the following restrictions have been caused by chest pain, chest tightness and angina:

	Severely restricted	Moderately restricted	Mildly restricted	Slightly restricted	Unrestricted	Limited for other reasons
Dress yourself	□1	□2	□3	□4	□5	□6
Indoor walk	□1	□2	□3	□4	□5	□6
Take a shower	□1	□2	□3	□4	□5	□6
Climbing or stairway (three floors, non-stop)	□1	□2	□3	□4	□5	□6
Outdoor activities or pick up	□1	□2	□3	□4	□5	□6
Walk lightly (one kilometer)	□1	□2	□3	□4	□5	□6
Jogging (one kilometer)	□1	□2	□3	□4	□5	□6
Lift or move heavy objects	□1	□2	□3	□4	□5	□6
Strenuous exercise (such as swimming or playing)	□1	□2	□3	□4	□5	□6

2. Compared with 4 weeks ago, when doing the maximum intensity of activity, chest pain, chest tightness and angina pectoris:
□1 obviously increase □2 Slight increase □3 the same □4 Minor reduction □5 obviously decrease

3. The average number of episodes of chest pain, chest tightness, and angina in the past 4 weeks:

□1 ≥four times/day □2 0ne-three times/day □3≥three times/week □4 one-two times/week

□5 < 1 time/week □6 no episode

4. The average number of nitro drugs (such as nitroglycerin) in chest pain, chest tightness, and angina pectoris over the past 4 weeks:

□1 ≥four times/day □2 0ne-three times/day □3≥three times/week □4 one-two times/week

□5 < 1 time/week □6 no episode

5. Due to chest pain, chest tightness and angina pectoris, follow the doctor's advice:

□1 Serious □2 Moderate □3 Minor □4 Very little □5 No □6 Doctor did not administer

6. Satisfaction with various measures for treating chest pain, chest tightness and angina:

□1 Dissatisfied □2 Most dissatisfied □3 Partially satisfied □4 Most satisfied □5 Highly satisfied

7. Satisfaction with the doctor's explanation for chest pain, chest tightness and angina:

□1 Dissatisfied □2 Most dissatisfied □3 Partially satisfied □4 Most satisfied □5 Highly satisfied

8. Overall, satisfaction with the current treatment of chest pain, chest tightness and angina:

□1 Dissatisfied □2 Most dissatisfied □3 Partially satisfied □4 Most satisfied □5 Highly satisfied

Visit4<br>20Week±4Day	Drug number |___|___|___|	Patient initials |___|___|___|___|	Date of visit<br>201|__|Year|__|__|Month|__|__|Day

9. In the past 4 weeks, chest pain, chest tightness and angina have affected the level of fun in life:

□1 Dissatisfied □2 Most dissatisfied □3 Partially satisfied □4 Most satisfied □5 Highly satisfied

10. If you have chest pain, chest tightness and angina in your future life, how would you feel?

□1 Dissatisfied □2 Most dissatisfied □3Partially satisfied □4 Most satisfied □5 Highly satisfied

11. Concerns about heart attack and sudden death:

□1 Always worried □2 Frequently worried □3 Sometimes worried □4 Very little worried □5 Never worry

* The Seattle Angina Scale is divided into 5 items and 19 items: Physical activity limitation (PL, question l), angina stable state (AS, question 2), angina pectoris (AF, question 3-4), treatment satisfaction (TS), question 5-8), disease cognition (DP, question 9-11). After scoring the 19 items of the 5 major items one by one, each score is forwarded.The standard score = (actual score - the lowest score in this aspect) / (the highest score in this aspect - the lowest score in this aspect) ✖100, the higher the score, the better the quality of life and the functional state of the patient.

Angina pectoris symptom score Score *: |___|___|___|

Number of angina attacks	□0 No	□2 2-6 episodes per week	□41-3 times a day	□6 more than 4 episodes per day
Angina duration	□0 No	□2 Each time the pain lasts less than or equal to 5 minutes	□4 Each time the pain lasts longer than 5 minutes and less than 10	□6 Each time the pain lasts for 10 minutes or more
Angina pain level	□0 No	□2 Physical activity that is heavier than daily activities causes angina and daily activities are asymptomatic. Activities that can cause angina, such as trotting on the ground, fast or holding heavy objects on the third floor, up steep slopes, etc.	□4 Daily physical activity causes angina and daily activities are slightly limited. Activities that can cause angina, such as walking 3-4 stations (3-4 miles) at normal speed under normal conditions, on the third floor, uphill, etc.	□6 Lighter physical activity than daily activities causes angina and daily activities are significantly limited. Activities that can cause angina, such as walking 1-2 stations (1-2 miles) at normal speed under normal conditions, on the second floor, gentle slope, etc.
Nitroglycerin dosage	□0 No	□2 1-4 tablets per week	□4 5-9tablets per week	□6 Take more than 10 tablets a week

Visit4<br>20Week±4Day	Drug number |___|___|___|	Patient initials |___|___|___|___|	Date of visit<br>201|__|Year|__|__|Month|__|__|Day

Laboratory inspection

Indicator	Measured Value	Unit	Clinical significance *<br>1 2 3 4	Anomalous cause explanation
**Blood sugar**		**Report date:** 201|___|Year|___|___|Month|___|___|Day		
FPG		mmol/L	□1 □2 □3 □4	
2hPG		mmol/L	□1 □2 □3 □4	
HbA1c		mg/dl	□1 □2 □3 □4	
Fasting insulin		μU/ml	□1 □2 □3 □4	
**Blood lipid**		**Report date:** 201|___|Year|___|___|Month|___|___|Day		
HDL-C		mmol/L	□1 □2 □3 □4	
LDL-C		mmol/L	□1 □2 □3 □4	
TG		mmol/L	□1 □2 □3 □4	
CH		mmol/L	□1 □2 □3 □4	
**C-reactive protein**		**Report date:** 201|___|Year|___|___|Month|___|___|Day		
CRP		mg/L	□1 □2 □3 □4	

* Clinical significance judgment: (1) normal (2) abnormal but no clinical significance (3) abnormal and clinical significance (4) not investigated.

Is there a combined medication?

□0No

□1Yes→Please fill out the “Combined Medication Record Form” on page 47.

Are there any adverse events?

□0No

□1Yes→Please fill out the “Adverse Event Record Form” on page 48 in detail.

Is there an unscheduled laboratory check?

□0No

□1Yes→Record the results to “Unplanned Laboratory Inspection Results” on page 49

Release of test drug: Ginkgo biloba drop pills 900 pills (10 bottles)

Researcher's signature: ____________________ **Date**: 201|___|Year|___|___|Month|___|___|Day

Visit5<br>28Week±4Day	Drug number<br>|___|___|___|	Patient initials<br>|___|___|___|___|	Date of visit<br>201|__|Year|__|__|Month|__|__|Day

Seattle Angina Questionnaire(SAQ) Score *：|___|___|___|

1. In the past 4 weeks, the following restrictions have been caused by chest pain, chest tightness and angina:

	Severely restricted	Moderately restricted	Mildly restricted	Slightly restricted	Unrestricted	Limited for other reasons
Dress yourself	□1	□2	□3	□4	□5	□6
Indoor walk	□1	□2	□3	□4	□5	□6
Take a shower	□1	□2	□3	□4	□5	□6
Climbing or stairway (three floors, non-stop)	□1	□2	□3	□4	□5	□6
Outdoor activities or pick up	□1	□2	□3	□4	□5	□6
Walk lightly (one kilometer)	□1	□2	□3	□4	□5	□6
Jogging (one kilometer)	□1	□2	□3	□4	□5	□6
Lift or move heavy objects	□1	□2	□3	□4	□5	□6
Strenuous exercise (such as swimming or playing)	□1	□2	□3	□4	□5	□6

2. Compared with 4 weeks ago, when doing the maximum intensity of activity, chest pain, chest tightness and angina pectoris:
□1 obviously increase □2 Slight increase □3 the same □4 Minor reduction □5 obviously decrease

3. The average number of episodes of chest pain, chest tightness, and angina in the past 4 weeks:

□1 ≥four times/day □2 0ne-three times/day □3≥three times/week □4 one-two times/week

□5 < 1 time/week □6 no episode

4. The average number of nitro drugs (such as nitroglycerin) in chest pain, chest tightness, and angina pectoris over the past 4 weeks:

□1 ≥four times/day □2 0ne-three times/day □3≥three times/week □4 one-two times/week

□5 < 1 time/week □6 no episode

5. Due to chest pain, chest tightness and angina pectoris, follow the doctor's advice:

□1 Serious □2 Moderate □3 Minor □4 Very little □5 No □6 Doctor did not administer

6. Satisfaction with various measures for treating chest pain, chest tightness and angina:

□1 Dissatisfied □2 Most dissatisfied □3 Partially satisfied □4 Most satisfied □5 Highly satisfied

7. Satisfaction with the doctor's explanation for chest pain, chest tightness and angina:

□1 Dissatisfied □2 Most dissatisfied □3 Partially satisfied □4 Most satisfied □5 Highly satisfied

8. Overall, satisfaction with the current treatment of chest pain, chest tightness and angina:

□1 Dissatisfied □2 Most dissatisfied □3 Partially satisfied □4 Most satisfied □5 Highly satisfied

9. In the past 4 weeks, chest pain, chest tightness and angina have affected the level of fun in life:

□$_1$ Dissatisfied □$_2$ Most dissatisfied □$_3$ Partially satisfied □$_4$ Most satisfied □$_5$ Highly satisfied

10. If you have chest pain, chest tightness and angina in your future life, how would you feel?

□$_1$ Dissatisfied □$_2$ Most dissatisfied □$_3$Partially satisfied □$_4$ Most satisfied □$_5$ Highly satisfied

11. Concerns about heart attack and sudden death:

□$_1$ Always worried □$_2$ Frequently worried □$_3$ Sometimes worried □$_4$ Very little worried □$_5$ Never worry

* The Seattle Angina Scale is divided into 5 items and 19 items: Physical activity limitation (PL, question l), angina stable state (AS, question 2), angina pectoris (AF, question 3-4), treatment satisfaction (TS), question 5-8), disease cognition (DP, question 9-11). After scoring the 19 items of the 5 major items one by one, each score is forwarded.The standard score = (actual score - the lowest score in this aspect) / (the highest score in this aspect - the lowest score in this aspect) ✖100, the higher the score, the better the quality of life and the functional state of the patient.

Angina pectoris symptom score Score *: |___|___|___|

Number of angina attacks	□$_0$ No	□$_2$ 2-6 episodes per week	□$_4$1-3 times a day	□$_6$ more than 4 episodes per day
Angina duration	□$_0$ No	□$_2$ Each time the pain lasts less than or equal to 5 minutes	□$_4$ Each time the pain lasts longer than 5 minutes and less than 10	□$_6$ Each time the pain lasts for 10 minutes or more
Angina pain level	□$_0$ No	□$_2$ Physical activity that is heavier than daily activities causes angina and daily activities are asymptomatic. Activities that can cause angina, such as trotting on the ground, fast or holding heavy objects on the third floor, up steep slopes, etc.	□$_4$ Daily physical activity causes angina and daily activities are slightly limited. Activities that can cause angina, such as walking 3-4 stations (3-4 miles) at normal speed under normal conditions, on the third floor, uphill, etc.	□$_6$ Lighter physical activity than daily activities causes angina and daily activities are significantly limited. Activities that can cause angina, such as walking 1-2 stations (1-2 miles) at normal speed under normal conditions, on the second floor, gentle slope, etc.
Nitroglycerin dosage	□$_0$ No	□$_2$ 1-4 tablets per week	□$_4$ 5-9tablets per week	□$_6$ Take more than 10 tablets a week

Visit5<br>28Week±4Day	Drug number<br>|___|___|___|	Patient initials<br>|___|___|___|___|	Date of visit<br>201|__|Year|__|__|Month|__|__|Day

Laboratory inspection

Indicator	Measured Value	Unit	Clinical significance *<br>1 2 3 4	Anomalous cause explanation
**Blood sugar**		**Report date:** 201|___|Year|___|___|Month|___|___|Day		
FPG		mmol/L	□1 □2 □3 □4	
2hPG		mmol/L	□1 □2 □3 □4	
HbA1c		mg/dl	□1 □2 □3 □4	
Fasting insulin		μU/ml	□1 □2 □3 □4	
**Blood lipid**		**Report date:** 201|___|Year|___|___|Month|___|___|Day		
HDL-C		mmol/L	□1 □2 □3 □4	
LDL-C		mmol/L	□1 □2 □3 □4	
TG		mmol/L	□1 □2 □3 □4	
CH		mmol/L	□1 □2 □3 □4	
**C-reactive protein**		**Report date:** 201|___|Year|___|___|Month|___|___|Day		
CRP		mg/L	□1 □2 □3 □4	

* Clinical significance judgment: (1) normal (2) abnormal but no clinical significance (3) abnormal and clinical significance (4) not investigated.

Visit5<br>28Week±4Day	Drug number<br>|___|___|___|	Patient initials<br>|___|___|___|___|	Date of visit<br>201|__|Year|__|__|Month|__|__|Day

Is the test drug returned?

□0No→Unreturned reason： □2 Take all □3 Lost □4 Other___________
□1Yes→ Ginkgo biloba drop pills |___|___|___| pills

Is there a combined medication?

□0No
□1Yes→Please fill out the "Combined Medication Record Form" on page 47.

Are there any adverse events?

□0No
□1Yes→Please fill out the "Adverse Event Record Form" on page 48 in detail.

Is there an unscheduled laboratory check?

□0No
□1Yes→Record the results to "Unplanned Laboratory Inspection Results" on page 49

Researcher's signature： ____________________**Date**： 201|___|Year|___|___|Month|___|___|Day

Visit6<br>30Week±4Day	Drug number<br>|___|___|___|	Patient initials<br>|___|___|___|___|	Date of visit<br>201|__|Year|__|__|Month|__|__|Day

Seattle Angina Questionnaire(SAQ) Score *：|___|___|___|

1. In the past 4 weeks, the following restrictions have been caused by chest pain, chest tightness and angina:

	Severely restricted	Moderately restricted	Mildly restricted	Slightly restricted	Unrestricted	Limited for other reasons
Dress yourself	□1	□2	□3	□4	□5	□6
Indoor walk	□1	□2	□3	□4	□5	□6
Take a shower	□1	□2	□3	□4	□5	□6
Climbing or stairway (three floors, non-stop)	□1	□2	□3	□4	□5	□6
Outdoor activities or pick up	□1	□2	□3	□4	□5	□6
Walk lightly (one kilometer)	□1	□2	□3	□4	□5	□6
Jogging (one kilometer)	□1	□2	□3	□4	□5	□6
Lift or move heavy objects	□1	□2	□3	□4	□5	□6
Strenuous exercise (such as swimming or playing)	□1	□2	□3	□4	□5	□6

2. Compared with 4 weeks ago, when doing the maximum intensity of activity, chest pain, chest tightness and angina pectoris:
□1 obviously increase □2 Slight increase □3 the same □4 Minor reduction □5 obviously decrease

3. The average number of episodes of chest pain, chest tightness, and angina in the past 4 weeks:

□1 ≥four times/day □2 0ne-three times/day □3≥three times/week □4 one-two times/week

□5 < 1 time/week □6 no episode

4. The average number of nitro drugs (such as nitroglycerin) in chest pain, chest tightness, and angina pectoris over the past 4 weeks:

□1 ≥four times/day □2 0ne-three times/day □3≥three times/week □4 one-two times/week

□5 < 1 time/week □6 no episode

5. Due to chest pain, chest tightness and angina pectoris, follow the doctor's advice:

□1 Serious □2 Moderate □3 Minor □4 Very little □5 No □6 Doctor did not administer

6. Satisfaction with various measures for treating chest pain, chest tightness and angina:

□1 Dissatisfied □2 Most dissatisfied □3 Partially satisfied □4 Most satisfied □5 Highly satisfied

7. Satisfaction with the doctor's explanation for chest pain, chest tightness and angina:

□1 Dissatisfied □2 Most dissatisfied □3 Partially satisfied □4 Most satisfied □5 Highly satisfied

8. Overall, satisfaction with the current treatment of chest pain, chest tightness and angina:

□1 Dissatisfied □2 Most dissatisfied □3 Partially satisfied □4 Most satisfied □5 Highly satisfied

9. In the past 4 weeks, chest pain, chest tightness and angina have affected the level of fun in life:

□$_1$ Dissatisfied □$_2$ Most dissatisfied □$_3$ Partially satisfied □$_4$ Most satisfied □$_5$ Highly satisfied

10. If you have chest pain, chest tightness and angina in your future life, how would you feel?

□$_1$ Dissatisfied □$_2$ Most dissatisfied □$_3$Partially satisfied □$_4$ Most satisfied □$_5$ Highly satisfied

11. Concerns about heart attack and sudden death:

□$_1$ Always worried □$_2$ Frequently worried □$_3$ Sometimes worried □$_4$ Very little worried □$_5$ Never worry

* The Seattle Angina Scale is divided into 5 items and 19 items: Physical activity limitation (PL, question l), angina stable state (AS, question 2), angina pectoris (AF, question 3-4), treatment satisfaction (TS), question 5-8), disease cognition (DP, question 9-11). After scoring the 19 items of the 5 major items one by one, each score is forwarded.The standard score = (actual score - the lowest score in this aspect) / (the highest score in this aspect - the lowest score in this aspect) ✖100, the higher the score, the better the quality of life and the functional state of the patient.

Angina pectoris symptom score Score *: |___|___|___|

Number of angina attacks	□$_0$ No	□$_2$ 2-6 episodes per week	□$_4$1-3 times a day	□$_6$ more than 4 episodes per day
Angina duration	□$_0$ No	□$_2$ Each time the pain lasts less than or equal to 5 minutes	□$_4$ Each time the pain lasts longer than 5 minutes and less than 10	□$_6$ Each time the pain lasts for 10 minutes or more
Angina pain level	□$_0$ No	□$_2$ Physical activity that is heavier than daily activities causes angina and daily activities are asymptomatic. Activities that can cause angina, such as trotting on the ground, fast or holding heavy objects on the third floor, up steep slopes, etc.	□$_4$ Daily physical activity causes angina and daily activities are slightly limited. Activities that can cause angina, such as walking 3-4 stations (3-4 miles) at normal speed under normal conditions, on the third floor, uphill, etc.	□$_6$ Lighter physical activity than daily activities causes angina and daily activities are significantly limited. Activities that can cause angina, such as walking 1-2 stations (1-2 miles) at normal speed under normal conditions, on the second floor, gentle slope, etc.
Nitroglycerin dosage	□$_0$ No	□$_2$ 1-4 tablets per week	□$_4$ 5-9tablets per week	□$_6$ Take more than 10 tablets a week

Visit6 30Week±4Day	Drug number |___|___|___|	Patient initials |___|___|___|___|	Date of visit 201|__|Year|__|__|Month|__|__|Day

Laboratory inspection

Indicator	Measured Value	Unit	Clinical significance * 1 2 3 4	Anomalous cause explanation
**Blood sugar**		**Report date：** 201|___|Year|___|___|Month|___|___|Day		
FPG		mmol/L	□1 □2 □3 □4	
2hPG		mmol/L	□1 □2 □3 □4	
HbA1c		mg/dl	□1 □2 □3 □4	
Fasting insulin		μU/ml	□1 □2 □3 □4	
**Blood lipid**		**Report date：** 201|___|Year|___|___|Month|___|___|Day		
HDL-C		mmol/L	□1 □2 □3 □4	
LDL-C		mmol/L	□1 □2 □3 □4	
TG		mmol/L	□1 □2 □3 □4	
CH		mmol/L	□1 □2 □3 □4	
**C-reactive protein**		**Report date：** 201|___|Year|___|___|Month|___|___|Day		
CRP		㎎/L	□1 □2 □3 □4	

* Clinical significance judgment: (1) normal (2) abnormal but no clinical significance (3) abnormal and clinical significance (4) not investigated.

Is there a combined medication?

□0No

□1Yes→Please fill out the “Combined Medication Record Form” on page 47.

Are there any adverse events?

□0No

□1Yes→Please fill out the “Adverse Event Record Form” on page 48 in detail.

Is there an unscheduled laboratory check?

□0No

□1Yes→Record the results to “Unplanned Laboratory Inspection Results” on page 49

Release of test drug: Ginkgo biloba drop pills 900 pills (10 bottles)

Researcher's signature: ____________________ **Date**: 201\|___\|Year\|___\|___\|Month\|___\|___\|Day

Visit7 38Week±4Day	Drug number |___|___|___|	Patient initials |___|___|___|___|	Date of visit 201|__|Year|__|__|Month|__|__|Day

Seattle Angina Questionnaire(SAQ) Score *：|___|___|___|

1. In the past 4 weeks, the following restrictions have been caused by chest pain, chest tightness and angina:

	Severely restricted	Moderately restricted	Mildly restricted	Slightly restricted	Unrestricted	Limited for other reasons
Dress yourself	□1	□2	□3	□4	□5	□6
Indoor walk	□1	□2	□3	□4	□5	□6
Take a shower	□1	□2	□3	□4	□5	□6
Climbing or stairway (three floors, non-stop)	□1	□2	□3	□4	□5	□6
Outdoor activities or pick up	□1	□2	□3	□4	□5	□6
Walk lightly (one kilometer)	□1	□2	□3	□4	□5	□6
Jogging (one kilometer)	□1	□2	□3	□4	□5	□6
Lift or move heavy objects	□1	□2	□3	□4	□5	□6
Strenuous exercise (such as swimming or playing)	□1	□2	□3	□4	□5	□6

2. Compared with 4 weeks ago, when doing the maximum intensity of activity, chest pain, chest tightness and angina pectoris:
□1 obviously increase □2 Slight increase □3 the same □4 Minor reduction □5 obviously decrease

3. The average number of episodes of chest pain, chest tightness, and angina in the past 4 weeks:

□1 ≥four times/day □2 0ne-three times/day □3≥three times/week □4 one-two times/week

□5 < 1 time/week □6 no episode

4. The average number of nitro drugs (such as nitroglycerin) in chest pain, chest tightness, and angina pectoris over the past 4 weeks:

□1 ≥four times/day □2 0ne-three times/day □3≥three times/week □4 one-two times/week

□5 < 1 time/week □6 no episode

5. Due to chest pain, chest tightness and angina pectoris, follow the doctor's advice:

□1 Serious □2 Moderate □3 Minor □4 Very little □5 No □6 Doctor did not administer

6. Satisfaction with various measures for treating chest pain, chest tightness and angina:

□1 Dissatisfied □2 Most dissatisfied □3 Partially satisfied □4 Most satisfied □5 Highly satisfied

7. Satisfaction with the doctor's explanation for chest pain, chest tightness and angina:

□1 Dissatisfied □2 Most dissatisfied □3 Partially satisfied □4 Most satisfied □5 Highly satisfied

8. Overall, satisfaction with the current treatment of chest pain, chest tightness and angina:

□1 Dissatisfied □2 Most dissatisfied □3 Partially satisfied □4 Most satisfied □5 Highly satisfied

9. In the past 4 weeks, chest pain, chest tightness and angina have affected the level of fun in life:

□$_1$ Dissatisfied □$_2$ Most dissatisfied □$_3$ Partially satisfied □$_4$ Most satisfied □$_5$ Highly satisfied

10. If you have chest pain, chest tightness and angina in your future life, how would you feel?

□$_1$ Dissatisfied □$_2$ Most dissatisfied □$_3$Partially satisfied □$_4$ Most satisfied □$_5$ Highly satisfied

11. Concerns about heart attack and sudden death:

□$_1$ Always worried □$_2$ Frequently worried □$_3$ Sometimes worried □$_4$ Very little worried □$_5$ Never worry

* The Seattle Angina Scale is divided into 5 items and 19 items: Physical activity limitation (PL, question l), angina stable state (AS, question 2), angina pectoris (AF, question 3-4), treatment satisfaction (TS), question 5-8), disease cognition (DP, question 9-11). After scoring the 19 items of the 5 major items one by one, each score is forwarded.The standard score = (actual score - the lowest score in this aspect) / (the highest score in this aspect - the lowest score in this aspect) ✖100, the higher the score, the better the quality of life and the functional state of the patient.

Angina pectoris symptom score Score *: |___|___|___|

Number of angina attacks	□$_0$ No	□$_2$ 2-6 episodes per week	□$_4$1-3 times a day	□$_6$ more than 4 episodes per day
Angina duration	□$_0$ No	□$_2$ Each time the pain lasts less than or equal to 5 minutes	□$_4$ Each time the pain lasts longer than 5 minutes and less than 10	□$_6$ Each time the pain lasts for 10 minutes or more
Angina pain level	□$_0$ No	□$_2$ Physical activity that is heavier than daily activities causes angina and daily activities are asymptomatic. Activities that can cause angina, such as trotting on the ground, fast or holding heavy objects on the third floor, up steep slopes, etc.	□$_4$ Daily physical activity causes angina and daily activities are slightly limited. Activities that can cause angina, such as walking 3-4 stations (3-4 miles) at normal speed under normal conditions, on the third floor, uphill, etc.	□$_6$ Lighter physical activity than daily activities causes angina and daily activities are significantly limited. Activities that can cause angina, such as walking 1-2 stations (1-2 miles) at normal speed under normal conditions, on the second floor, gentle slope, etc.
Nitroglyceri n dosage	□$_0$ No	□$_2$ 1-4 tablets per week	□$_4$ 5-9tablets per week	□$_6$ Take more than 10 tablets a week

Visit7 38Week±4Day	Drug number	Patient initials	Date of visit
	|___|___|___|	|___|___|___|___|	201|__|Year|__|__|Month|__|__|Day

Laboratory inspection

Indicator	Measured Value	Unit	Clinical significance * 1 2 3 4	Anomalous cause explanation
**Blood sugar**		**Report date:** 201|___|Year|___|___|Month|___|___|Day		
FPG		mmol/L	□1 □2 □3 □4	
2hPG		mmol/L	□1 □2 □3 □4	
HbA1c		mg/dl	□1 □2 □3 □4	
Fasting insulin		µU/ml	□1 □2 □3 □4	
**Blood lipid**		**Report date:** 201|___|Year|___|___|Month|___|___|Day		
HDL-C		mmol/L	□1 □2 □3 □4	
LDL-C		mmol/L	□1 □2 □3 □4	
TG		mmol/L	□1 □2 □3 □4	
CH		mmol/L	□1 □2 □3 □4	
**C-reactive protein**		**Report date:** 201|___|Year|___|___|Month|___|___|Day		
CRP		mg/L	□1 □2 □3 □4	

* Clinical significance judgment: (1) normal (2) abnormal but no clinical significance (3) abnormal and clinical significance (4) not investigated.

Visit7<br>38Week±4Day	Drug number<br>|___|___|___|	Patient initials<br>|___|___|___|___|	Date of visit<br>201|__|Year|__|__|Month|__|__|Day

Is the test drug returned?

□$_0$No→Unreturned reason: □$_2$ Take all □$_3$ Lost □$_4$ Other___________

□$_1$Yes→ Ginkgo biloba drop pills |___|___|___| pills

Is there a combined medication?

□$_0$No

□$_1$Yes→Please fill out the "Combined Medication Record Form" on page 47.

Are there any adverse events?

□$_0$No

□$_1$Yes→Please fill out the "Adverse Event Record Form" on page 48 in detail.

Is there an unscheduled laboratory check?

□$_0$No

□$_1$Yes→Record the results to "Unplanned Laboratory Inspection Results" on page 49

Researcher's signature: ____________________**Date**: 201|___|Year|___|___|Month|___|___|Day

Visit8<br>40Week±4Day	Drug number<br>|___|___|___|	Patient initials<br>|___|___|___|___|	Date of visit<br>201|__|Year|__|__|Month|__|__|Day

Seattle Angina Questionnaire(SAQ) Score *：|___|___|___|

1. In the past 4 weeks, the following restrictions have been caused by chest pain, chest tightness and angina:

	Severely restricted	Moderately restricted	Mildly restricted	Slightly restricted	Unrestricted	Limited for other reasons
Dress yourself	□1	□2	□3	□4	□5	□6
Indoor walk	□1	□2	□3	□4	□5	□6
Take a shower	□1	□2	□3	□4	□5	□6
Climbing or stairway (three floors, non-stop)	□1	□2	□3	□4	□5	□6
Outdoor activities or pick up	□1	□2	□3	□4	□5	□6
Walk lightly (one kilometer)	□1	□2	□3	□4	□5	□6
Jogging (one kilometer)	□1	□2	□3	□4	□5	□6
Lift or move heavy objects	□1	□2	□3	□4	□5	□6
Strenuous exercise (such as swimming or playing)	□1	□2	□3	□4	□5	□6

2. Compared with 4 weeks ago, when doing the maximum intensity of activity, chest pain, chest tightness and angina pectoris:
□1 obviously increase □2 Slight increase □3 the same □4 Minor reduction □5 obviously decrease

3. The average number of episodes of chest pain, chest tightness, and angina in the past 4 weeks:

□1 ≥four times/day □2 0ne-three times/day □3≥three times/week □4 one-two times/week

□5 < 1 time/week □6 no episode

4. The average number of nitro drugs (such as nitroglycerin) in chest pain, chest tightness, and angina pectoris over the past 4 weeks:

□1 ≥four times/day □2 0ne-three times/day □3≥three times/week □4 one-two times/week

□5 < 1 time/week □6 no episode

5. Due to chest pain, chest tightness and angina pectoris, follow the doctor's advice:

□1 Serious □2 Moderate □3 Minor □4 Very little □5 No □6 Doctor did not administer

6. Satisfaction with various measures for treating chest pain, chest tightness and angina:

□1 Dissatisfied □2 Most dissatisfied □3 Partially satisfied □4 Most satisfied □5 Highly satisfied

7. Satisfaction with the doctor's explanation for chest pain, chest tightness and angina:

□1 Dissatisfied □2 Most dissatisfied □3 Partially satisfied □4 Most satisfied □5 Highly satisfied

8. Overall, satisfaction with the current treatment of chest pain, chest tightness and angina:

□1 Dissatisfied □2 Most dissatisfied □3 Partially satisfied □4 Most satisfied □5 Highly satisfied

9. In the past 4 weeks, chest pain, chest tightness and angina have affected the level of fun in life:

□1 Dissatisfied □2 Most dissatisfied □3 Partially satisfied □4 Most satisfied □5 Highly satisfied

10. If you have chest pain, chest tightness and angina in your future life, how would you feel?

□1 Dissatisfied □2 Most dissatisfied □3Partially satisfied □4 Most satisfied □5 Highly satisfied

11. Concerns about heart attack and sudden death:

□1 Always worried □2 Frequently worried □3 Sometimes worried □4 Very little worried □5 Never worry

* The Seattle Angina Scale is divided into 5 items and 19 items: Physical activity limitation (PL, question l), angina stable state (AS, question 2), angina pectoris (AF, question 3-4), treatment satisfaction (TS), question 5-8), disease cognition (DP, question 9-11). After scoring the 19 items of the 5 major items one by one, each score is forwarded.The standard score = (actual score - the lowest score in this aspect) / (the highest score in this aspect - the lowest score in this aspect) ✖100, the higher the score, the better the quality of life and the functional state of the patient.

Angina pectoris symptom score   Score *: |___|___|___|

Number of angina attacks	□0 No	□2 2-6 episodes per week	□41-3 times a day	□6 more than 4 episodes per day
Angina duration	□0 No	□2 Each time the pain lasts less than or equal to 5 minutes	□4 Each time the pain lasts longer than 5 minutes and less than 10	□6 Each time the pain lasts for 10 minutes or more
Angina pain level	□0 No	□2 Physical activity that is heavier than daily activities causes angina and daily activities are asymptomatic. Activities that can cause angina, such as trotting on the ground, fast or holding heavy objects on the third floor, up steep slopes, etc.	□4 Daily physical activity causes angina and daily activities are slightly limited. Activities that can cause angina, such as walking 3-4 stations (3-4 miles) at normal speed under normal conditions, on the third floor, uphill, etc.	□6 Lighter physical activity than daily activities causes angina and daily activities are significantly limited. Activities that can cause angina, such as walking 1-2 stations (1-2 miles) at normal speed under normal conditions, on the second floor, gentle slope, etc.
Nitroglycerin dosage	□0 No	□2 1-4 tablets per week	□4 5-9tablets per week	□6 Take more than 10 tablets a week

Visit8 40Week±4Day	Drug number |___|___|___|	Patient initials |___|___|___|___|	Date of visit 201|__|Year|__|__|Month|__|__|Day

Laboratory inspection

Indicator	Measured Value	Unit	Clinical significance * 1 2 3 4	Anomalous cause explanation
**Blood sugar**		**Report date：** 201|___|Year|___|___|Month|___|___|Day		
FPG		mmol/L	□1 □2 □3 □4	
2hPG		mmol/L	□1 □2 □3 □4	
HbA1c		mg/dl	□1 □2 □3 □4	
Fasting insulin		μU/ml	□1 □2 □3 □4	
**Blood lipid**		**Report date：** 201|___|Year|___|___|Month|___|___|Day		
HDL-C		mmol/L	□1 □2 □3 □4	
LDL-C		mmol/L	□1 □2 □3 □4	
TG		mmol/L	□1 □2 □3 □4	
CH		mmol/L	□1 □2 □3 □4	
**C-reactive protein**		**Report date：** 201|___|Year|___|___|Month|___|___|Day		
CRP		mg/L	□1 □2 □3 □4	

* Clinical significance judgment: (1) normal (2) abnormal but no clinical significance (3) abnormal and clinical significance (4) not investigated.

Is there a combined medication?

□0No

□1Yes→Please fill out the “Combined Medication Record Form” on page 47.

Are there any adverse events?

□0No

□1Yes→Please fill out the “Adverse Event Record Form” on page 48 in detail.

Is there an unscheduled laboratory check?

□0No

□1Yes→Record the results to “Unplanned Laboratory Inspection Results” on page 49

Release of test drug: Ginkgo biloba drop pills 900 pills (10 bottles)

Researcher's signature: ____________________**Date**: 201|___|Year|___|___|Month|___|___|Day

Seattle Angina Questionnaire(SAQ) Score *：|__|__|__|

1. In the past 4 weeks, the following restrictions have been caused by chest pain, chest tightness and angina:

	Severely restricted	Moderately restricted	Mildly restricted	Slightly restricted	Unrestricted	Limited for other reasons
Dress yourself	□1	□2	□3	□4	□5	□6
Indoor walk	□1	□2	□3	□4	□5	□6
Take a shower	□1	□2	□3	□4	□5	□6
Climbing or stairway (three floors, non-stop)	□1	□2	□3	□4	□5	□6
Outdoor activities or pick up	□1	□2	□3	□4	□5	□6
Walk lightly (one kilometer)	□1	□2	□3	□4	□5	□6
Jogging (one kilometer)	□1	□2	□3	□4	□5	□6
Lift or move heavy objects	□1	□2	□3	□4	□5	□6
Strenuous exercise (such as swimming or playing)	□1	□2	□3	□4	□5	□6

2. Compared with 4 weeks ago, when doing the maximum intensity of activity, chest pain, chest tightness and angina pectoris:
□1 obviously increase □2 Slight increase □3 the same □4 Minor reduction □5 obviously decrease

3. The average number of episodes of chest pain, chest tightness, and angina in the past 4 weeks:

□1 ≥four times/day □2 0ne-three times/day □3≥three times/week □4 one-two times/week

□5 < 1 time/week □6 no episode

4. The average number of nitro drugs (such as nitroglycerin) in chest pain, chest tightness, and angina pectoris over the past 4 weeks:

□1 ≥four times/day □2 0ne-three times/day □3≥three times/week □4 one-two times/week

□5 < 1 time/week □6 no episode

5. Due to chest pain, chest tightness and angina pectoris, follow the doctor's advice:

□1 Serious □2 Moderate □3 Minor □4 Very little □5 No □6 Doctor did not administer

6. Satisfaction with various measures for treating chest pain, chest tightness and angina:

□1 Dissatisfied □2 Most dissatisfied □3 Partially satisfied □4 Most satisfied □5 Highly satisfied

7. Satisfaction with the doctor's explanation for chest pain, chest tightness and angina:

□1 Dissatisfied □2 Most dissatisfied □3 Partially satisfied □4 Most satisfied □5 Highly satisfied

8. Overall, satisfaction with the current treatment of chest pain, chest tightness and angina:

□1 Dissatisfied □2 Most dissatisfied □3 Partially satisfied □4 Most satisfied □5 Highly satisfied

9. In the past 4 weeks, chest pain, chest tightness and angina have affected the level of fun in life:

□1 Dissatisfied □2 Most dissatisfied □3 Partially satisfied □4 Most satisfied □5 Highly satisfied

10. If you have chest pain, chest tightness and angina in your future life, how would you feel?

□1 Dissatisfied □2 Most dissatisfied □3Partially satisfied □4 Most satisfied □5 Highly satisfied

11. Concerns about heart attack and sudden death:

□1 Always worried □2 Frequently worried □3 Sometimes worried □4 Very little worried □5 Never worry

* The Seattle Angina Scale is divided into 5 items and 19 items: Physical activity limitation (PL, question l), angina stable state (AS, question 2), angina pectoris (AF, question 3-4), treatment satisfaction (TS), question 5-8), disease cognition (DP, question 9-11). After scoring the 19 items of the 5 major items one by one, each score is forwarded.The standard score = (actual score - the lowest score in this aspect) / (the highest score in this aspect - the lowest score in this aspect) ✖100, the higher the score, the better the quality of life and the functional state of the patient.

Angina pectoris symptom score Score *: |___|___|___|

Number of angina attacks	□0 No	□2 2-6 episodes per week	□41-3 times a day	□6 more than 4 episodes per day
Angina duration	□0 No	□2 Each time the pain lasts less than or equal to 5 minutes	□4 Each time the pain lasts longer than 5 minutes and less than 10	□6 Each time the pain lasts for 10 minutes or more
Angina pain level	□0 No	□2 Physical activity that is heavier than daily activities causes angina and daily activities are asymptomatic. Activities that can cause angina, such as trotting on the ground, fast or holding heavy objects on the third floor, up steep slopes, etc.	□4 Daily physical activity causes angina and daily activities are slightly limited. Activities that can cause angina, such as walking 3-4 stations (3-4 miles) at normal speed under normal conditions, on the third floor, uphill, etc.	□6 Lighter physical activity than daily activities causes angina and daily activities are significantly limited. Activities that can cause angina, such as walking 1-2 stations (1-2 miles) at normal speed under normal conditions, on the second floor, gentle slope, etc.
Nitroglycerin dosage	□0 No	□2 1-4 tablets per week	□4 5-9tablets per week	□6 Take more than 10 tablets a week

Visit9 48Week±4Day	Drug number	Patient initials	Date of visit
	|___|___|___|	|___|___|___|___|	201|__|Year|__|__|Month|__|__|Day

Laboratory inspection

Indicator	Measured Value	Unit	Clinical significance * 1 2 3 4	Anomalous cause explanation
**Blood sugar**		**Report date:** 201|___|Year|___|___|Month|___|___|Day		
FPG		mmol/L	□1 □2 □3 □4	
2hPG		mmol/L	□1 □2 □3 □4	
HbA1c		mg/dl	□1 □2 □3 □4	
Fasting insulin		μU/ml	□1 □2 □3 □4	
**Blood lipid**		**Report date:** 201|___|Year|___|___|Month|___|___|Day		
HDL-C		mmol/L	□1 □2 □3 □4	
LDL-C		mmol/L	□1 □2 □3 □4	
TG		mmol/L	□1 □2 □3 □4	
CH		mmol/L	□1 □2 □3 □4	
**C-reactive protein**		**Report date:** 201|___|Year|___|___|Month|___|___|Day		
CRP		mg/L	□1 □2 □3 □4	

* Clinical significance judgment: (1) normal (2) abnormal but no clinical significance (3) abnormal and clinical significance (4) not investigated.

Visit9<br>48Week±4Day	Drug number<br>|___|___|___|	Patient initials<br>|___|___|___|___|	Date of visit<br>201|__|Year|__|__|Month|__|__|Day

Is the test drug returned?

□$_0$No→Unreturned reason：□$_2$ Take all □$_3$ Lost □$_4$ Other___________

□$_1$Yes→ Ginkgo biloba drop pills |___|___|___| pills

Is there a combined medication?

□$_0$No

□$_1$Yes→Please fill out the “Combined Medication Record Form” on page 47.

Are there any adverse events?

□$_0$No

□$_1$Yes→Please fill out the “Adverse Event Record Form” on page 48 in detail.

Is there an unscheduled laboratory check?

□$_0$No

□$_1$Yes→Record the results to “Unplanned Laboratory Inspection Results” on page 49

Researcher's signature：____________________**Date**：201|___|Year|___|___|Month|___|___|Day

Visit10<br>50Week±4Day	Drug number<br>|___|___|___|	Patient initials<br>|___|___|___|___|	Date of visit<br>201|__|Year|__|__|Month|__|__|Day

Seattle Angina Questionnaire(SAQ) Score *：|___|___|___|

1. In the past 4 weeks, the following restrictions have been caused by chest pain, chest tightness and angina:

	Severely restricted	Moderately restricted	Mildly restricted	Slightly restricted	Unrestricted	Limited for other reasons
Dress yourself	□1	□2	□3	□4	□5	□6
Indoor walk	□1	□2	□3	□4	□5	□6
Take a shower	□1	□2	□3	□4	□5	□6
Climbing or stairway (three floors, non-stop)	□1	□2	□3	□4	□5	□6
Outdoor activities or pick up	□1	□2	□3	□4	□5	□6
Walk lightly (one kilometer)	□1	□2	□3	□4	□5	□6
Jogging (one kilometer)	□1	□2	□3	□4	□5	□6
Lift or move heavy objects	□1	□2	□3	□4	□5	□6
Strenuous exercise (such as swimming or playing)	□1	□2	□3	□4	□5	□6

2. Compared with 4 weeks ago, when doing the maximum intensity of activity, chest pain, chest tightness and angina pectoris:
□1 obviously increase □2 Slight increase □3 the same □4 Minor reduction □5 obviously decrease

3. The average number of episodes of chest pain, chest tightness, and angina in the past 4 weeks:

□1 ≥four times/day □2 0ne-three times/day □3≥three times/week □4 one-two times/week

□5 < 1 time/week □6 no episode

4. The average number of nitro drugs (such as nitroglycerin) in chest pain, chest tightness, and angina pectoris over the past 4 weeks:

□1 ≥four times/day □2 0ne-three times/day □3≥three times/week □4 one-two times/week

□5 < 1 time/week □6 no episode

5. Due to chest pain, chest tightness and angina pectoris, follow the doctor's advice:

□1 Serious □2 Moderate □3 Minor □4 Very little □5 No □6 Doctor did not administer

6. Satisfaction with various measures for treating chest pain, chest tightness and angina:

□1 Dissatisfied □2 Most dissatisfied □3 Partially satisfied □4 Most satisfied □5 Highly satisfied

7. Satisfaction with the doctor's explanation for chest pain, chest tightness and angina:

□1 Dissatisfied □2 Most dissatisfied □3 Partially satisfied □4 Most satisfied □5 Highly satisfied

8. Overall, satisfaction with the current treatment of chest pain, chest tightness and angina:

□1 Dissatisfied □2 Most dissatisfied □3 Partially satisfied □4 Most satisfied □5 Highly satisfied

9. In the past 4 weeks, chest pain, chest tightness and angina have affected the level of fun in life:

□$_1$ Dissatisfied □$_2$ Most dissatisfied □$_3$ Partially satisfied □$_4$ Most satisfied □$_5$ Highly satisfied

10. If you have chest pain, chest tightness and angina in your future life, how would you feel?

□$_1$ Dissatisfied □$_2$ Most dissatisfied □$_3$Partially satisfied □$_4$ Most satisfied □$_5$ Highly satisfied

11. Concerns about heart attack and sudden death:

□$_1$ Always worried □$_2$ Frequently worried □$_3$ Sometimes worried □$_4$ Very little worried □$_5$ Never worry

* The Seattle Angina Scale is divided into 5 items and 19 items: Physical activity limitation (PL, question l), angina stable state (AS, question 2), angina pectoris (AF, question 3-4), treatment satisfaction (TS), question 5-8), disease cognition (DP, question 9-11). After scoring the 19 items of the 5 major items one by one, each score is forwarded.The standard score = (actual score - the lowest score in this aspect) / (the highest score in this aspect - the lowest score in this aspect) ✖100, the higher the score, the better the quality of life and the functional state of the patient.

Angina pectoris symptom score Score *: |___|___|___|

Number of angina attacks	□$_0$ No	□$_2$ 2-6 episodes per week	□$_4$1-3 times a day	□$_6$ more than 4 episodes per day
Angina duration	□$_0$ No	□$_2$ Each time the pain lasts less than or equal to 5 minutes	□$_4$ Each time the pain lasts longer than 5 minutes and less than 10	□$_6$ Each time the pain lasts for 10 minutes or more
Angina pain level	□$_0$ No	□$_2$ Physical activity that is heavier than daily activities causes angina and daily activities are asymptomatic. Activities that can cause angina, such as trotting on the ground, fast or holding heavy objects on the third floor, up steep slopes, etc.	□$_4$ Daily physical activity causes angina and daily activities are slightly limited. Activities that can cause angina, such as walking 3-4 stations (3-4 miles) at normal speed under normal conditions, on the third floor, uphill, etc.	□$_6$ Lighter physical activity than daily activities causes angina and daily activities are significantly limited. Activities that can cause angina, such as walking 1-2 stations (1-2 miles) at normal speed under normal conditions, on the second floor, gentle slope, etc.
Nitroglycerin dosage	□$_0$ No	□$_2$ 1-4 tablets per week	□$_4$ 5-9tablets per week	□$_6$ Take more than 10 tablets a week

Visit10<br>50Week±4Day	Drug number<br>|___|___|___|	Patient initials<br>|___|___|___|___|	Date of visit<br>201|__|Year|__|__|Month|__|__|Day

Laboratory inspection

Indicator	Measured Value	Unit	Clinical significance * 1 2 3 4	Anomalous cause explanation
**Blood sugar**		**Report date:** 201|___|Year|___|___|Month|___|___|Day		
FPG		mmol/L	□1 □2 □3 □4	
2hPG		mmol/L	□1 □2 □3 □4	
HbA1c		mg/dl	□1 □2 □3 □4	
Fasting insulin		μU/ml	□1 □2 □3 □4	
**Blood lipid**		**Report date:** 201|___|Year|___|___|Month|___|___|Day		
HDL-C		mmol/L	□1 □2 □3 □4	
LDL-C		mmol/L	□1 □2 □3 □4	
TG		mmol/L	□1 □2 □3 □4	
CH		mmol/L	□1 □2 □3 □4	
**C-reactive protein**		**Report date:** 201|___|Year|___|___|Month|___|___|Day		
CRP		mg/L	□1 □2 □3 □4	

* Clinical significance judgment: (1) normal (2) abnormal but no clinical significance (3) abnormal and clinical significance (4) not investigated.

Is there a combined medication?

□0No

□1Yes→Please fill out the "Combined Medication Record Form" on page 47.

Are there any adverse events?

□0No

□1Yes→Please fill out the "Adverse Event Record Form" on page 48 in detail.

Is there an unscheduled laboratory check?

□0No

□1Yes→Record the results to "Unplanned Laboratory Inspection Results" on page 49

Release of test drug: Ginkgo biloba drop pills 900 pills (10 bottles)

Researcher's signature: ____________________ **Date**: 201|___|Year|___|___|Month|___|___|Day

Visit11<br>58Week±4Day	Drug number<br>|___|___|___|	Patient initials<br>|___|___|___|___|	Date of visit<br>201|__|Year|__|__|Month|__|__|Day

Vital signs

Body temperature (℃)	Breathe (Times/minute)	Heart rate (Times/minute)	Blood pressure (mmHg)
|___|___|.|___|	|___|___|	|___|___|___|	|___|___|___|/|___|___|___|

Seattle Angina Questionnaire(SAQ) Score *：\|___\|___\|___\|

1. In the past 4 weeks, the following restrictions have been caused by chest pain, chest tightness and angina:

	Severely restricted	Moderately restricted	Mildly restricted	Slightly restricted	Unrestricted	Limited for other reasons
Dress yourself	□1	□2	□3	□4	□5	□6
Indoor walk	□1	□2	□3	□4	□5	□6
Take a shower	□1	□2	□3	□4	□5	□6
Climbing or stairway (three floors, non-stop)	□1	□2	□3	□4	□5	□6
Outdoor activities or pick up	□1	□2	□3	□4	□5	□6
Walk lightly (one kilometer)	□1	□2	□3	□4	□5	□6
Jogging (one kilometer)	□1	□2	□3	□4	□5	□6
Lift or move heavy objects	□1	□2	□3	□4	□5	□6
Strenuous exercise (such as swimming or playing)	□1	□2	□3	□4	□5	□6

2. Compared with 4 weeks ago, when doing the maximum intensity of activity, chest pain, chest tightness and angina pectoris:
□1 obviously increase □2 Slight increase □3 the same □4 Minor reduction □5 obviously decrease

3. The average number of episodes of chest pain, chest tightness, and angina in the past 4 weeks:

□1 ≥four times/day □2 0ne-three times/day □3≥three times/week □4 one-two times/week

□5 < 1 time/week □6 no episode

4. The average number of nitro drugs (such as nitroglycerin) in chest pain, chest tightness, and angina pectoris over the past 4 weeks:

□1 ≥four times/day □2 0ne-three times/day □3≥three times/week □4 one-two times/week

□5 < 1 time/week □6 no episode

5. Due to chest pain, chest tightness and angina pectoris, follow the doctor's advice:

□1 Serious □2 Moderate □3 Minor □4 Very little □5 No □6 Doctor did not administer

6. Satisfaction with various measures for treating chest pain, chest tightness and angina:

□1 Dissatisfied □2 Most dissatisfied □3 Partially satisfied □4 Most satisfied □5 Highly satisfied

7. Satisfaction with the doctor's explanation for chest pain, chest tightness and angina:

□1 Dissatisfied □2 Most dissatisfied □3 Partially satisfied □4 Most satisfied □5 Highly satisfied

8. Overall, satisfaction with the current treatment of chest pain, chest tightness and angina:

□1 Dissatisfied □2 Most dissatisfied □3 Partially satisfied □4 Most satisfied □5 Highly satisfied

9. In the past 4 weeks, chest pain, chest tightness and angina have affected the level of fun in life:

□1 Dissatisfied □2 Most dissatisfied □3 Partially satisfied □4 Most satisfied □5 Highly satisfied

10. If you have chest pain, chest tightness and angina in your future life, how would you feel?

□1 Dissatisfied □2 Most dissatisfied □3Partially satisfied □4 Most satisfied □5 Highly satisfied

11. Concerns about heart attack and sudden death:

□1 Always worried □2 Frequently worried □3 Sometimes worried □4 Very little worried □5 Never worry

* The Seattle Angina Scale is divided into 5 items and 19 items: Physical activity limitation (PL, question l), angina stable state (AS, question 2), angina pectoris (AF, question 3-4), treatment satisfaction (TS), question 5-8), disease cognition (DP, question 9-11). After scoring the 19 items of the 5 major items one by one, each score is forwarded.The standard score = (actual score - the lowest score in this aspect) / (the highest score in this aspect - the lowest score in this aspect) ✖100, the higher the score, the better the quality of life and the functional state of the patient.

Angina pectoris symptom score Score *: |___|___|___|

Number of angina attacks	□0 No	□2 2-6 episodes per week	□41-3 times a day	□6 more than 4 episodes per day
Angina duration	□0 No	□2 Each time the pain lasts less than or equal to 5 minutes	□4 Each time the pain lasts longer than 5 minutes and less than 10	□6 Each time the pain lasts for 10 minutes or more
Angina pain level	□0 No	□2 Physical activity that is heavier than daily activities causes angina and daily activities are asymptomatic. Activities that can cause angina, such as trotting on the ground, fast or holding heavy objects on the third floor, up steep slopes, etc.	□4 Daily physical activity causes angina and daily activities are slightly limited. Activities that can cause angina, such as walking 3-4 stations (3-4 miles) at normal speed under normal conditions, on the third floor, uphill, etc.	□6 Lighter physical activity than daily activities causes angina and daily activities are significantly limited. Activities that can cause angina, such as walking 1-2 stations (1-2 miles) at normal speed under normal conditions, on the second floor, gentle slope, etc.
Nitroglycerin dosage	□0 No	□2 1-4 tablets per week	□4 5-9tablets per week	□6 Take more than 10 tablets a week

Laboratory inspection

Indicator	Measured Value	Unit	Clinical significance * 1 2 3 4	Anomalous cause explanation
**Blood routine**		**Report date:** 201|___|Year|___|___|Month|___|___|Day		
HGB		g/L	□1 □2 □3 □4	
RBC		$\times 10^{12}$L	□1 □2 □3 □4	
WBC		$\times 10^{9}$/L	□1 □2 □3 □4	
PLT		$\times 10^{9}$/L	□1 □2 □3 □4	
**Urine routine**		**Report date:** 201|___|Year|___|___|Month|___|___|Day		
Urine PH			□1 □2 □3 □4	
PRO		—	□1 □2 □3 □4	
ERY		/HPF	□**1** □**2** □**3** □**4**	
White blood cells（LEU）		/HPF	□**1** □**2** □**3** □**4**	
**Liver and kidney function**		**Report date:** 201|___|Year|___|___|Month|___|___|Day		
ALT		U/L	□1 □2 □3 □4	
AST		U/L	□1 □2 □3 □4	
ALP		U/L	□1 □2 □3 □4	
TBIL		μmol/L	□1 □2 □3 □4	
γ-GT		U/L	□1 □2 □3 □4	
TBA		μmol/L	□1 □2 □3 □4	
BUN		mmol/L	□1 □2 □3 □4	
Cr		μmol/L	□1 □2 □3 □4	
**blood sugar**		**Report date:** 201|___|Year|___|___|Month|___|___|Day		
FPG		mmol/L	□1 □2 □3 □4	
2hPG		mmol/L	□1 □2 □3 □4	
HbA1c		mg/dl	□1 □2 □3 □4	
Fasting insulin		μU/ml	□1 □2 □3 □4	

Visit11 58Week±4Day	Drug number |___|___|___|	Patient initials |___|___|___|___|	Date of visit 201|__|Year|__|__|Month|__|__|Day

**Blood lipid**		**Report date:** 201|___|Year|___|___|Month|___|___|Day		
HDL-C		mmol/L	□1 □2 □3 □4	
LDL-C		mmol/L	□1 □2 □3 □4	
TG		mmol/L	□1 □2 □3 □4	
CH		mmol/L	□1 □2 □3 □4	
**C-reactive protein**		**Report date:** 201|___|Year|___|___|Month|___|___|Day		
CRP		mg/L	□1 □2 □3 □4	
**Electrocardiogram**		**Report date:** 201|___|Year|___|___|Month|___|___|Day		
ECG		—	□1 □2 □3 □4	

* Clinical significance judgment: (1) normal (2) abnormal but no clinical significance (3) abnormal and clinical significance (4) not investigated.

Is the test drug returned?

□0No→Unreturned reason: □2 Take all □3 Lost □4 Other___________

□1Yes→ Ginkgo biloba drop pills |___|___|___| pills

Is there a combined medication?

□0No

□1Yes→Please fill out the “Combined Medication Record Form” on page 47.

Are there any adverse events?

□0No

□1Yes→Please fill out the “Adverse Event Record Form” on page 48 in detail.

Is there an unscheduled laboratory check?

□0No

□1Yes→Record the results to “Unplanned Laboratory Inspection Results” on page 49

Researcher's signature: ____________________**Date**: 201|___|Year|___|___|Month|___|___|Day

Drug number	Patient initials	Date of visit
|___|___|___|	|___|___|___|___|	201|__|Year|__|__|Month|__|__|Day

Combined medication record

Are there any combined medications during the trial? □$_0$ No □$_1$ Yes→ please fill in the form below

Drug Name	Indications or medication reasons	Single dose	Unit (01-11)	Number of doses (01-08)	Route of administ ration (01-10)	Start medication date	Still using	End of medication date
			| | |	| | |	| | |	|__|__|__|__| Year|__|__|Month|__|__|Day	□$_1$ Yes □$_0$ No	|__|__|__|__| Year|__|__|Month|__|__|Day
			| | |	| | |	| | |	|__|__|__|__| Year|__|__|Month|__|__|Day	□$_1$ Yes □$_0$ No	|__|__|__|__| Year|__|__|Month|__|__|Day
			| | |	| | |	| | |	|__|__|__|__| Year|__|__|Month|__|__|Day	□$_1$ Yes □$_0$ No	|__|__|__|__| Year|__|__|Month|__|__|Day
			| | |	| | |	| | |	|__|__|__|__| Year|__|__|Month|__|__|Day	□$_1$ Yes □$_0$ No	|__|__|__|__| Year|__|__|Month|__|__|Day
			| | |	| | |	| | |	|__|__|__|__| Year|__|__|Month|__|__|Day	□$_1$ Yes □$_0$ No	|__|__|__|__| Year|__|__|Month|__|__|Day
			| | |	| | |	| | |	|__|__|__|__| Year|__|__|Month|__|__|Day	□$_1$ Yes □$_0$ No	|__|__|__|__| Year|__|__|Month|__|__|Day
			| | |	| | |	| | |	|__|__|__|__| Year|__|__|Month|__|__|Day	□$_1$ Yes □$_0$ No	|__|__|__|__| Year|__|__|Month|__|__|Day

Unit code	Daily dosing code	Route of administration
01=microgram 07=Capsule<br>02=milligram 08=piece<br>03=g 04=ml 09=drops<br>05=milliequivalents 11=Others<br>06=internationalunit	01=1 time per day 05=Use when necessary<br>02=2 times a day 06=Use every other day<br>03=3 times a day 08=Others<br>04=4 times a day<br>07=Use before going to bed	01=Oral 07=inhalation inhalation<br>02 = vein 08=Personal use<br>03=intramuscular injection 09=Local use<br>04=Subcutaneous injection 10 = other<br>05=Intradermal injection<br>06 = inhalation

Researcher's signature: ________________________ **Date**: 201\|___\|Year\|___\|___\|Month\|___\|___\|Day

Drug number	Patient initials	Date of visit
|___|___|___|	|___|___|___|___|	201|__|Year|__|__|Month|__|__|Day

Adverse event record

Are there any adverse events during the trial? □$_0$ No □$_1$ Yes → please fill in the form below

Adverse event name	Date of occurrence	Severity 1-3	Relationship with test drugs 1-5	Measures taken for test drugs 1-5	Ending 1-5	End date	Whether serious adverse events	Is there a combined medication?
	|__|__|__|__| Year|__|__|Month|__|__|Day	|__|	|__|	|__|	|__|	|__|__|__|__| Year|__|__|Month|__|__|Day	□$_1$ Yes □$_0$ No	□$_1$ Yes □$_0$ No
	|__|__|__|__| Year|__|__|Month|__|__|Day	|__|	|__|	|__|	|__|	|__|__|__|__| Year|__|__|Month|__|__|Day	□$_1$ Yes □$_0$ No	□$_1$ Yes □$_0$ No
	|__|__|__|__| Year|__|__|Month|__|__|Day	|__|	|__|	|__|	|__|	|__|__|__|__| Year|__|__|Month|__|__|Day	□$_1$ Yes □$_0$ No	□$_1$ Yes □$_0$ No
	|__|__|__|__| Year|__|__|Month|__|__|Day	|__|	|__|	|__|	|__|	|__|__|__|__| Year|__|__|Month|__|__|Day	□$_1$ Yes □$_0$ No	□$_1$ Yes □$_0$ No
**Severity coding (according to CTCAE3.0)**<br>**1=mild**<br>**2=moderate**<br>**3=severe**	**Coding with test drug**<br>**1= definitely relevant**<br>**2=may be relevant**<br>**3=may not be relevant**<br>**4= definitely has nothing to do**<br>**5=Unable to determine**	**Code of action for the test drug**<br>**1=no action taken**<br>**2=reduce the dose**<br>**3=increase the dose**<br>**4=suspension of treatment**<br>**5=permanent termination of treatment**					**Ending code**<br>**1=recovery**<br>**2=mitigation**<br>**3=continuous**<br>**4=death**<br>**5=unknown**	

Researcher's signature: ________________________ **Date**: 201\|___\|Year\|___\|___\|Month\|___\|___\|Day

Drug number	Patient initials	Date of visit
|___|___|___|	|___|___|___|___|	201|__|Year|__|__|Month|__|__|Day

Unplanned laboratory test results

Is there an unscheduled laboratory test during the trial? □$_0$No □$_1$Yes→ please fill in the form below

Check date（yyyymmdd）	Check item	Examination result (marked unit)	Check the reason	Clinical significance *	Whether it has an impact on this study
				1 2 3 4	
				□$_1$ □$_2$ □$_3$ □$_4$	□$_1$ Yes □$_0$ No
				□$_1$ □$_2$ □$_3$ □$_4$	□$_1$ Yes □$_0$ No
				□$_1$ □$_2$ □$_3$ □$_4$	□$_1$ Yes □$_0$ No
				□$_1$ □$_2$ □$_3$ □$_4$	□$_1$ Yes □$_0$ No
				□$_1$ □$_2$ □$_3$ □$_4$	□$_1$ Yes □$_0$ No
				□$_1$ □$_2$ □$_3$ □$_4$	□$_1$ Yes □$_0$ No
				□$_1$ □$_2$ □$_3$ □$_4$	□$_1$ Yes □$_0$ No
				□$_1$ □$_2$ □$_3$ □$_4$	□$_1$ Yes □$_0$ No
				□$_1$ □$_2$ □$_3$ □$_4$	□$_1$ Yes □$_0$ No
				□$_1$ □$_2$ □$_3$ □$_4$	□$_1$ Yes □$_0$ No
				□$_1$ □$_2$ □$_3$ □$_4$	□$_1$ Yes □$_0$ No
				□$_1$ □$_2$ □$_3$ □$_4$	□$_1$ Yes □$_0$ No
				□$_1$ □$_2$ □$_3$ □$_4$	□$_1$ Yes □$_0$ No
				□$_1$ □$_2$ □$_3$ □$_4$	□$_1$ Yes □$_0$ No
				□$_1$ □$_2$ □$_3$ □$_4$	□$_1$ Yes □$_0$ No
				□$_1$ □$_2$ □$_3$ □$_4$	□$_1$ Yes □$_0$ No

* Clinical significance judgment: (1) normal (2) abnormal but no clinical significance (3) abnormal and clinical significance (4) not investigated.

Researcher's signature: __________________**Date**: 201\|___\|Year\|___\|___\|Month\|___\|___\|Day

Drug number	Patient initials	Date of visit
|___|___|___|	|___|___|___|___|	201|__|Year|__|__|Month|__|__|Day

Medication compliance

Should take the dose: Ginkgo biloba drop pills/Ginkgo biloba drop pills simulation agent 5040 pills (56 bottles)<br>Actual dose: |___|___|___|Pills; lost doses |___|___|___|Pills.<br>Compliance with medication = actual dose / dose of medication ×100% = |___|___|. |___|___|%

Summary of test completion

Subject's first medication date： 201|__|Year|__|__|Month|__|__|Day<br>Subject's last medication date： 201|__|Year|__|__|Month|__|__|Day<br>Does the subject have a concomitant medication during the trial? □$_0$No □$_1$Yes→Please fill in the "Consolidated Drug Record Form"<br>Did the subject have an adverse event during the trial? □$_0$No □$_1$Yes→Please fill in the "Adverse Event Record Form"<br>Did the subject have serious adverse events during the trial? □$_0$No □$_1$Yes→Please fill out the "Serious Adverse Event Report Form"<br>Did the subject complete a clinical trial? □$_1$Yes □$_0$No →Please fill out the form below	
**Patient Suspension Test Date:** 201|__|Year|__|__|Month|__|__|Day	
**The first to propose a suspension test is: (please choose one)**<br>□$_1$ patient<br>□$_2$ test researcher<br>□$_3$ sponsor<br>□$_4$ other:	**The main reason for the suspension test is: (please choose one)**<br>□$_1$ adverse events (insulated adverse events table)<br>□$_2$ lack of efficacy<br>□$_3$ violation of the test plan<br>□$_4$ lost<br>□$_5$ subject withdraws informed consent<br>□$_6$ other reasons:

Researcher's signature:____________________**Date:**201\|___\|Year\|___\|___\|Month\|___\|___\|Day

Lead researcher review statement
**I confirmed that the pages of the patient's case report form signed by me have been checked by me and confirmed that all information is true, accurate and in accordance with the research protocol requirements.**<br>**Signature of the main investigator:**<br>**Date:** 201|__|Year|__|__|Month|__|__|Day

Project		Observation period											
		Baseline (-7-0d)	Visit1 (8week ±4day)	Visit 2 （10week±4day）	Visit 3 （18week±4day）	Visit 4 （20week±4day）	Visit 5 (28week±4day)	Visit 6 (30week±4day)	Visit 7 (38week±4day)	Visit 8 (40week±4day)	Visit 9 (48week±4day)	Visit 10 (50week±4day)	Visit 11 (58week ±4day)
Signing informed consent		√											
Inclusion/exclusion criteria		√											
Medical history / demographic data		√											
Laboratory inspection	blood sugar	√	√	√	√	√	√	√	√	√	√	√	√
	Blood lipid	√	√	√	√	√	√	√	√	√	√	√	√
	CRP	√	√	√	√	√	√	√	√	√	√	√	√
	Blood routine	√	√										√
	Urine routine	√	√										√
	liver function	√	√										√
	Renal function	√	√										√
	Urine pregnancy test	√											
vital	Body temperature,	√	√										√

signs	breathing, heart rate												
	blood pressure	√	√										√
Physical examination		√											√
12-lead ECG		√	√										√
Angina pectoris symptom score		√	√	√	√	√	√	√	√	√	√	√	√
Seattle Angina Questionnaire		√	√	√	√	√	√	√	√	√	√	√	√
AE record			√	√	√	√	√	√	√	√	√	√	√
combined medication/treatment record		√	√	√	√	√	√	√	√	√	√	√	√
Distribute drugs		√		√		√		√		√		√	
Recover remaining test drugs and count			√		√		√		√		√		√
Clinical efficacy assessment													√
