# Supplementary material for: Efficacy and Safety of Ginkgo Biloba Pills for Coronary Heart Disease with Impaired Glucose Regulation: Study Protocol for a Series of N-of-1 Randomized, Double-Blind, Placebo-Controlled Trials
Source: Evid Based Complement Alternat Med. 2018 Oct 14;2018:7571629. doi: 10.1155/2018/7571629 (PMC6204161; doi:10.1155/2018/7571629)
Supplement: Supplementary 4 — Additional file 4: Informed consent form. [file 7571629.f4.pdf]

Informed Consent Form • Informed Notice Page

Dear patient: We invite you to participate in a single-case randomized controlled trial of Ginkgo Biloba in the treatment of coronary heart disease with impaired glucose regulation. When your research doctor or researcher and you discuss the informed consent form, you can ask a professional to explain and discuss where you have questions. We encourage you to communicate fully with your family and friends before making a decision. If you are participating in research on other projects, please inform your research doctor or professional in advance.

1. Research background and research purposes

With the development of China's economy and living standards, the prevalence and mortality of coronary atherosclerotic heart disease (CHD) in China are significantly higher than before, and it is younger. The Global Burden of Disease report published in the Lancet in 2015 shows that in 2013, the cause of death in China was ranked second among coronary heart disease. Therefore, cardiovascular disease is a serious challenge for China's public health and health industry. At the 2004 European Society of Cardiology Annual Meeting, the European Heart Survey released the results of a survey on the state of glucose metabolism in patients with coronary heart disease. The studies have shown that approximately two-thirds of patients with coronary heart disease have diabetes or impaired glucose tolerance. The results of this study further demonstrate the intersection and coexistence of diabetes and cardiovascular disease, and also indicate that impaired glucose regulation is prevalent in patients with cardiovascular disease. The US Department of Healthcare Research and Quality conducted a systematic review of 156 pre-diabetes prognosis studies, suggesting a higher risk of developing diabetes and cardiovascular disease in pre-diabetes. Early identification and treatment of pre-diabetes patients may reduce or delay the progression of diabetes and its associated cardiovascular complications and microvascular disease. Therefore, effective measures should be taken in time to intervene in the pre-diabetes period.

Ginkgo biloba is a leaf of Ginkgo biloba L. Ginkgo biloba, which has attracted much attention due to its high medicinal value. In traditional Chinese medicine, Ginkgo biloba is considered to be flat, sweet, bitter, and astringent, has the function of promoting blood circulation and removing blood stasis, removing obstruction in the Channels to stop pain, astringe Lung-Qi and relieve cough and melting turbidity and reducing lipid decoction. It is commonly used in the treatment of obstruction of collaterals by blood stasis, chest stuffiness and pains, apoplectic hemiplegia, etc. Related studies have shown that the effective medicinal components extracted from Ginkgo biloba are mainly flavonoids, terpene lactone, and organic acids, which increase blood flow, prevent blood clots, reduce blood lipids, regulate the central nervous system, improve memory function, and improve cognitive function, etc. The known pharmacological effects are mainly related to the mechanisms of antagonizing platelet activating factor (PAF), anti-oxidation, scavenging free radicals, and regulating the release of excitatory amino acids.

Metabolomics is mainly to estimate the physiological and pathological

mechanisms of the body by analyzing all metabolite changes and changes over time caused by external influences on the body (cell, tissue or organism). Metabolomics is at the bottom of system biology. Small molecule metabolism is the final result of the action of biological organisms. The analysis of metabolites of biological fluids can reflect the pathophysiological state of organisms more directly and accurately, and is often used diagnosis and efficacy evaluation in clinical research.

The single-case randomized controlled (N-of-1) trial is a clinical efficacy evaluation method that meets the self-regulation and characteristics of the individual medical treatment in traditional Chinese medicine (TCM). The study of N-of-1 for TCM can highlight individualized diagnosis and treatment and maintain TCM characteristics. It can also make the clinical research of TCM more scientific, and can avoid the influence of subjective selection, implementation and measurement bias, and individual differences, especially for chronic diseases requiring long-term treatment. Therefore, in this study, patients with CHD and IGR will be selected as the study subjects. N-of-1 trials will be used to evaluate the clinical efficacy of Ginkgo biloba pills and to explore the individualized therapeutic evaluation methods applicable to TCM research.

2. Inclusion of exclusion criteria

Inclusion criteria:

(1)Male and female patients with clear history of previous myocardial infarction or history of percutaneous coronary intervention(PCI)or history of coronary artery bypass grafting(CABG)(at least 3months or more),or who have coronary angiography or coronary CT angiography(CTA)results suggested at least one coronary artery stenosis and lumen stenosis ≥50%;

(2)In line with the criteria for stable angina, and the number of episodes of angina pectoris ≥ 2 times per week;

(3)Comply with the diagnostic criteria of blood stasis syndrome of coronary heart disease (CHD);

(4)Comply with the 2016 Diabetes Association (ADA) published criteria for impaired diagnosis of glucose regulation;

(5)Aged between 18 and 75 years;

(6)Participants voluntarily participated in this study, signed informed consent and had good compliance.

Exclusion criteria:

(1)With congenital or rheumatic heart disease or severe cardiopulmonary insufficiency (grade 3 and 4 of cardiac function),or uncontrolled severe arrhythmias (including ventricular tachycardia, supraventricular tachycardia),or not controlled hypertension (systolic blood pressure ≥ 160 mmHg or diastolic blood pressure ≥ 100 mmHg);

(2)With cerebrovascular disease, or with severe liver and kidney dysfunction, or with endocrine, urinary, blood system and other serious primary diseases;

(3) Within 4 weeks, there was history of major organ surgery such as head, chest or abdomen or bleeding tendency;

(4)Those who have taken hypo glycemic agents or glucocorticoids, thiazide

diuretics and other drugs that affect blood sugar levels within 3 months;

(5)People with diseases affecting blood glucose metabolism, such as thyroid glands and adrenal diseases, or those with previous history of the aforementioned diseases;

(6)Allergies or persons allergic to known ingredients of the study drug;

(7)Pregnancy and lactation women or those with a pregnancy plan;

(8)Subjects who participated in other clinical trials in the last 3 months;

(9)Researchers consider that subjects should not participate in clinical trials.

3. Research process

(1)How many people will participate in this research?

Twelve people will participate in this study in this hospital.

(2)What will be needed if I participate in the research?

The study adopts the principle of voluntary participation and meets the standard of admission. The age is between 18 and 75 years old and those who can cooperate with the examination and treatment can sign up.

(3)Treatment course and drug dosage

On the basis of basic treatment, Ginkgo biloba drop pills/Ginkgo biloba drop pill simulator, 5 pills/time, 3 times/day, taken in a random group cycle, 8 weeks in each period, 2 weeks before each period. This test cycle is 58 weeks.

If you agree to participate in this study, please sign an informed consent form. Before taking the medicine, the hospital will conduct a physical examination, blood test, urine test, and electrocardiogram to determine if you are suitable for the study. After confirming that you can participate in this study, you will be randomly assigned to the test group (take Ginkgo biloba drop pills) or the control group (take Ginkgo biloba drop pill simulator, that is, the appearance, smell, taste and therapeutic drugs are the same, but not effective Pharmaceutical ingredients). The entire study lasted for 58 weeks, with medication duration of 48 weeks. You will be admitted to the hospital for 8 weeks, 10 weeks, 18 weeks, 20 weeks, 28 weeks, 30 weeks, 38 weeks, 40 weeks, 48 weeks, 50 weeks and 58 weeks. You can opt out of the study at any time without any penalty, and you will not lose any benefits that you should have. However, if you decide to withdraw from the study during the study, we encourage you to consult with your doctor beforehand. Considering your safety issues, you may be scheduled to perform an inspection after you quit.

4. Possible benefits of participating in the study

Direct benefit: You will likely receive direct medical benefits such as improved condition.

Potential benefits: This study may cure the disease or prevent/mitigate the progression of the disease, but we cannot guarantee it. We hope that the information you get from this study will benefit your patients with the same condition in the future.

5. Participate in the study of possible risks

So far, no adverse reactions have occurred during the clinical application of this product after searching in the existing literature database. If you experience any discomfort or any unforeseen circumstances during the study, regardless of whether it

is related to the drug, you should promptly notify your doctor, who will make judgments and medical treatment.

Doctors and researchers will do their utmost to prevent possible harm from this study. If an adverse event occurs in a clinical study, the doctor will try to cure it, and the medical professional will identify whether it is related to the study drug. Determining the true sponsor (Wanbangde Pharmaceutical Group Co., Ltd.) will provide treatment costs and corresponding compensation for research-related damage. And this point has been stipulated in China's "Quality Management Specifications for Clinical Trials". You need to go to the hospital for follow-up on time during the study, which may cause you trouble or inconvenience.

6. Related expenses

Ginkgo biloba pills and laboratory tests related to the study taken during this study will be free of charge and you will not be responsible for additional costs. In addition, when you return to the hospital for 8 weeks, 10 weeks, 18 weeks, 20 weeks, 28 weeks, 30 weeks, 38 weeks, 40 weeks, 48 weeks, 50 weeks and 58 weeks, you will be provided with a transportation subsidy of 200 yuan each time.

7. Reasons to terminate your participation in the trial

(1)The investigator determines that conducting the test is harmful to you.

(2)There are important deviations in the design or implementation of clinical trial protocols, and it is difficult to evaluate drug effects.

(3)You have not taken the medicine according to the doctor's instructions.

(4)Serious adverse reactions occurred during the study.

(5)The applicant can suspend clinical trials in advance due to research funding and other reasons.

(6)Clinical trials were can celled by the State Food and Drug Administration/national authorities

In the event of the above, the investigator has the right to terminate your participation in the trial without your consent.

8. Is personal information confidential?

Everything about you, including your identity, medical history, condition, medical examination, and laboratory results, will be strictly confidential to the extent permitted by law. Only authorized researchers, ethics committees, and research projects can review your records, and the Food and Drug Administration is allowed to review your medical records related to the study to verify the authenticity and accuracy of the data collected by the Institute. It does not cover your personal details. Your name will not appear in any publicly available materials or reports related to this research.

9. Subject's rights and responsibilities

Your rights

You can refuse to participate in the study, or opt out of the study at any time during the study, without any reason. This decision will not affect your future treatment. If you participate in this study, you will be asked to sign this consent form, and you have the right to withdraw from the study at any stage of the study without discrimination or any unfair treatment, and your rights and corresponding medical

treatment will not be affected.

Your responsibility

As a subject, you need to provide real information about your medical history and current physical condition, inform the research doctor of any discomfort, and do not take any drugs, food, etc. that have been told that you are restricted. You need to tell the research doctor if you have recently participated in other research or are currently participating in other research.

10. What should I do now?

Thank you for reading the above materials. If you decide to participate in this study, please tell your doctor that he/she will arrange all the research for you.

Regardless of the research results, we will try our best to publish them.

Please keep this information.

Informed consent form •Consent signature page

Clinical Research Project Name: A single-case randomized controlled trial of Ginkgo Biloba in the treatment of coronary heart disease with impaired glucose regulation.

Sponsor: Wanbangde Pharmaceutical Group Co., Ltd.

Agree to the statement

I have read the above introduction to this study and have the opportunity to discuss and ask questions with the doctor about this study.

All the questions I raised were answered satisfactorily. I understand the risks and benefits that may arise from participating in this study. I know that participating in the study is voluntary. I confirm that I have enough time to consider it and understand that:

I can always ask your doctor for more information.

I can withdraw from the study at any time without discrimination or retaliation, and medical treatment and benefits will not be affected.

It is also clear that if I withdraw from the study, especially if I withdraw from the study due to drug reasons, if I tell the doctor about the changes in the condition, I will complete the corresponding physical examination and physical and chemical examination, which will be very beneficial to me and the whole study.

If you take any other medications for your illness, I will ask the doctor for advice beforehand or tell the doctor truthfully afterwards.

I agree with the drug regulatory authority, the ethics committee, or an authorized researcher to review my research.

I agree □ or refuse □ to use my medical records, blood samples and urine samples in addition to other experimental studies other than this study.

I will receive a copy of the signed and dated informed consent form.

Finally, I decided to agree to participate in this study and to ensure that I am as close as possible to my doctor's advice.

Patient signature: ____________ID number □□□□□□□□□□□□□□□□□□

Patient contact number: _________________ Date: ____ Years_______Month_______Day

Signature of legal representative:____________ID number □□□□□□□□□□□□□□□□□□

Contact number: _______________________Mobile number: _________

I confirm that the details of the study have been explained to the volunteers, including their powers and possible benefits and risks. And give him a copy of the signed informed consent.

Researcher's signature: ______ Date: ____ Years_______Month_______Day

Researcher's contact number: ________________________

Ethics Committee of Xiyuan Hospital, China Academy of Chinese Medical Sciences

Contact number: 010-62835646
